# Binarization of Metaheuristics: Is the Transfer Function Really Important?

**DOI:** 10.3390/biomimetics8050400

**Published:** 2023-09-01

**Authors:** José Lemus-Romani, Broderick Crawford, Felipe Cisternas-Caneo, Ricardo Soto, Marcelo Becerra-Rozas

**Affiliations:** 1 Escuela de Construcción Civil, Pontificia Universidad Católica de Chile, Avenida Vicuña Mackenna 4860, Macul, Santiago 7820436, Chile; 2 Escuela de Ingeniería Informática, Pontificia Universidad Católica de Valparaíso, Avenida Brasil 2241, Valparaíso 2362807, Chile; felipe.cisternas.c@mail.pucv.cl (F.C.-C.); ricardo.soto@pucv.cl (R.S.); marcelo.becerra.r@mail.pucv.cl (M.B.-R.)

**Keywords:** binarization scheme selection, grey wolf optimizer, sine cosine algorithm, whale optimization algorithm, set covering problem, Q-learning, diversity metrics

## Abstract

In this work, an approach is proposed to solve binary combinatorial problems using continuous metaheuristics. It focuses on the importance of binarization in the optimization process, as it can have a significant impact on the performance of the algorithm. Different binarization schemes are presented and a set of actions, which combine different transfer functions and binarization rules, under a selector based on reinforcement learning is proposed. The experimental results show that the binarization rules have a greater impact than transfer functions on the performance of the algorithms and that some sets of actions are statistically better than others. In particular, it was found that sets that incorporate the elite or elite roulette binarization rule are the best. Furthermore, exploration and exploitation were analyzed through percentage graphs and a statistical test was performed to determine the best set of actions. Overall, this work provides a practical approach for the selection of binarization schemes in binary combinatorial problems and offers guidance for future research in this field.

## 1. Introduction

In recent years, the optimization of systems has become a fundamental task in various areas of industry and technology. The search for optimal solutions to complex and multidimensional problems is a constant challenge in fields such as engineering, economics, physics, and computer science. In this sense, the application of metaheuristic optimization algorithms has become a valuable tool for finding efficient and effective solutions to these problems. Furthermore, the use of continuous metaheuristics to solve binary problems has become increasingly relevant due to their ability to find optimal solutions in a short period of time [1]. These techniques are capable of efficiently exploring and exploiting the search space, making them ideal for optimization problems in fields such as engineering, data science, and industry. However, it is important to note that the proper selection of parameters and search strategies is essential for obtaining optimal results.

The proposal of this work is to study different sets of actions (combinations of transfer functions and binarization rules) in order to evaluate the impact on the resolution of binary combinatorial problems, where different sets were evaluated in the binarization scheme selection (BSS) proposed in [2], where Q-learning, a machine learning technique, acts as an intelligent selector of binarization schemes.

The no free lunch (NFL) theorem [3] states that there is no single algorithm capable of reaching the optimal solution to all optimization problems. With this, researchers have the motivation to innovate and/or create new algorithms and validate them on different optimization problems with different domains. Tests were carried out with three good metaheuristics (MHs): grey wolf optimization [4], the whale optimization algorithm [5], and the sine cosine algorithm (SCA) [6].

The grey wolf optimizer has been used for example in feature selection [7], clustering applications [8], design and tuning controllers [9], power dispatch problems [10], economic dispatch problems [11], robotics and path planning [12], scheduling [13], and training neural networks [14].

The whale optimization algorithm has been used for example in optimal power flow problems [15], economic dispatch problems [16], electric vehicle charging station locating problems [17], image segmentation [18], feature selection [19], drug toxicity prediction [20], and CO${}_{2}$ emissions prediction and forecasting [21].

The sine cosine algorithm has been used for example in trajectory controller problems [22], feature selection [23], power management [24], network integration [25], engineering problems [26], and image processing [27].

The main contributions of this work are as follows:

- Evaluate different sets of transfer functions and binarization rules.
- Explore the importance of binarization rules compared to transfer functions.
- Compare the results in three different and complex metaheuristics.
- Conduct a comprehensive comparison of the results obtained by solving the set covering problem.

The structure of the content in the paper is as follows: In Section 2, a review of the related works on the use of continuous metaheuristics and reinforcement learning in combinatorial binary problems will be presented. In Section 2.1, we will present how continuous metaheuristics solve binary combinatorial optimization problems. In Section 2.4, hybridization between continuous metaheuristics and Q-learning will be presented, where Q-learning acts as an intelligence selector of binarization schemes. In Section 3, the different sets of actions (pairs of transfer functions and binarization rules) to be compared will be presented. In Section 4, the results obtained and the analysis performed will be presented, ending with the final conclusions in Section 5.

## 2. Related Work

In this section, we will discuss related work on four topics. The first topic is on continuous MHs for solving combinatorial problems, discussed in Section 2.1. To perform work in binary domains, the second topic is two-step techniques, covered in Section 2.2. Additionally, in Section 2.3, we will explain the MH used in this study. Finally, in Section 2.4, we describe the binarization schemes selector (BSS) hybridization technique.

### 2.1. Continuous Metaheuristics to Solve Combinatorial Problems

The binarization techniques used in continuous MHs involve transferring continuous domain values to binary domains, with the aim of maintaining the quality of moves and generating high-quality binary solutions. While some MHs operate on binary domains without a binary scheme, studies have demonstrated that continuous MHs supported by a binary scheme perform exceptionally well on multiple NP-hard combinatorial problems [1]. Examples of such MHs include the binary bat algorithm [28,29], particle swarm optimization [30], binary sine cosine algorithm [2,31,32,33], binary salp swarm algorithm [34,35], binary grey wolf optimizer [32,36,37], binary dragonfly algorithm [38,39], the binary whale optimization algorithm [2,32,40], and the binary magnetic optimization algorithm [41].

In the scientific literature, two main groups of binary schemes used to solve combinatorial problems can be identified. The first group refers to operators that do not cause alterations in the operations related to different elements of the MH. Within this group, two-step techniques stand out as the most widely used in recent years, as they are considered to be the most efficient in terms of convergence and their ability to find optimal solutions. These techniques are based on modifying the solution in the first step and discretizing it into a 0 or a 1 in the second step [42]. In addition, the angle modulation technique is also used in this group as it has been shown to be effective in solving combinatorial problems [43].

On the other hand, the second group of binary schemes includes methods that alter the normal operation of an MH. For example, the quantum binary approach, which is based on the application of quantum mechanisms to solve combinatorial problems [44]. In addition, also included in this group are set-based approaches, which focus on the selection of solution sets to improve the efficiency of the MH. Finally, clustering-based techniques, such as the k-means approach [45,46], are also considered in this second group, as they modify the normal operation of the MH to improve its ability to find optimal solutions.

### 2.2. Two-Step Techniques

In the scientific community, two-step binarization schemes are very relevant [1]. They have been widely used to solve a variety of combinatorial problems [47]. As the name suggests, this binarization scheme consists of two stages. The first stage involves the application of a transfer function [30], which transfers the values generated by the continuous MH to a continuous interval between 0 and 1. The second stage consists of the application of a binarization rule, which discretizes the numbers within that interval into binary values. This technique has been shown to be effective in solving combinatorial problems, since it allows the quality moves of the continuous MH to be preserved, while generating high-quality binary solutions.

#### 2.2.1. Transfer Function

**Figure 1:**
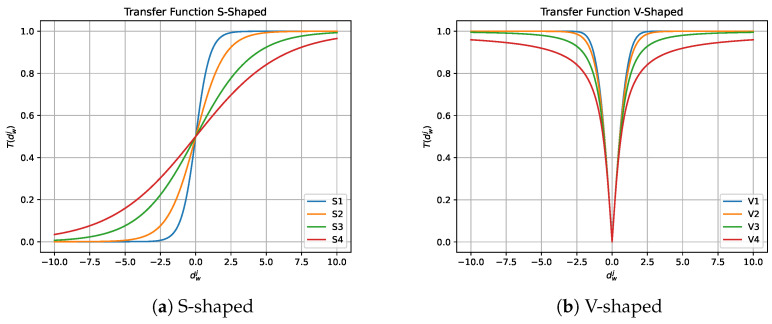
S-shaped and V-shaped transfer functions.

**Figure 2:**
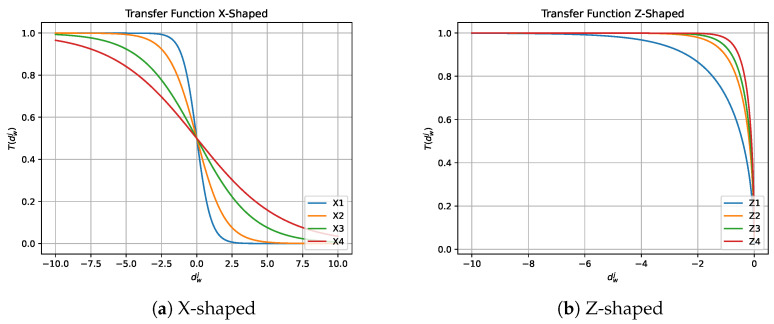
X-shaped and Z-shaped transfer functions.

In 1997, Kennedy et al. [48] introduced transfer functions in the field of optimization. Their main advantage is that they provide a probability between 0 and 1 with low computational cost. There are several types of transfer functions, including those in the form of an S [30,49] and a V [50], as well as in the forms of O, Z, X, Q, and U, among others. These functions are used to transfer values generated by the continuous MH to a continuous interval between 0 and 1, allowing the quality movements of the continuous MH to be preserved while high-quality binary solutions are generated. The families of transfer functions we have used in this work can be seen in Table 1 and Table 2 and Figure 1 and Figure 2. The notation $d_{w}^{j}$ observed in Table 1 and Table 2 corresponds to the continuous value of the *j*-th dimension of the *w*-th individual resulting from the perturbation performed by the continuous metaheuristic.

It is important to note that no transfer function is superior to the others in all cases in which they have been used, since according to the “no free lunch” theorem there is no universal optimization algorithm that is better than the others in all situations. Therefore, due to this theorem we have room for experimentation and analysis of new optimization algorithms.

On the other hand, different researchers [38,55,56,57] incorporated a parameter to these transfer functions, thus developing time-varying transfer functions. This parameter varies iteration by iteration, thus generating a new transfer function. For example, in [38], the parameter the authors incorporate is defined as follows:

(1) $$\tau =\left(1-\frac{t}{T}\right)\cdot \tau_{max}+\frac{t}{T}\cdot \tau_{min}$$

**Figure 3:**
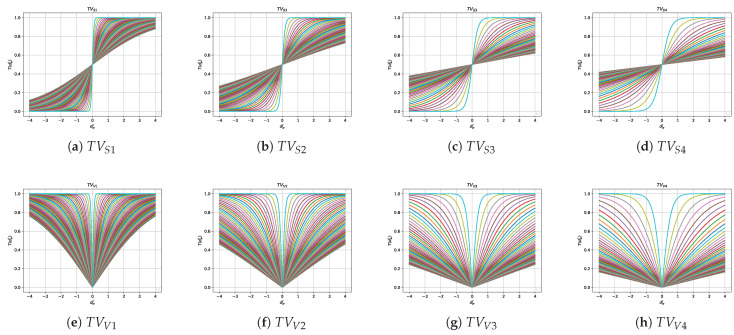
Time-varying S-shaped and V-shaped transfer functions when $\tau_{max}=4$ and $\tau_{min}=0.01$ during 100 iterations with time step 2 [38].

This parameter τ is added to the transfer functions of the S-shaped and V-shaped family. Table 3 shows the new time-varying transfer functions equation proposed by the authors and Figure 3 shows the results of running 100 iterations of each time-varying transfer function.

#### 2.2.2. Binarization Rule

The process of binarization involves converting continuous values into binary values, that is, values of 0 or 1. In this context, binarization rules are applied to the probability obtained from the transfer function to obtain a binary value. There are various different techniques described in the scientific literature [58] that can be utilized for this binarization process. Some of these techniques are illustrated in Table 4, and can vary depending on the context and specific project needs. It is crucial to consider appropriate use of the binarization technique to obtain accurate and reliable results.

The notation $X_{w}^{j}$ observed in Table 4 corresponds to the *j*-th dimension binary value of the *w*-th current individual and $X_{Best}^{j}$ corresponds to the *j*-th dimension binary value of the best solution.

### 2.3. Metaheuristics

Metaheuristics are general-purpose algorithms that provide good solutions in a reasonable time. The search process consists of balancing the diversification and intensification phases by means of operators specific to each algorithm [59]. Exploration aims to find tentative regions with good solutions and exploitation intensifies the search for the best regions to try to find better solutions.

Human behavior, genetic evolution, social behavior of animals, and physical phenomena are some of the main sources of inspiration for the authors, and every year new metaheuristics are developed based on the no free lunch theorem [3]. This theorem tells us that there is no supreme algorithm that solves all optimization problems.

In general, metaheuristics are designed and used to solve continuous optimization problems. In the following sections, we will present a brief summary of the three metaheuristics that have been used in this study with the aim of providing a background as to how the metaheuristic works; for more information see the seminal manuscripts.

#### 2.3.1. Sine Cosine Algorithm

The sine cosine algorithm (SCA) was proposed by Mirjalili [6]. This metaheuristic has two main equations combined into one (Equation (2)) and four main parameters for the position update of the solutions ($r_{1}$, $r_{2}$, $r_{3}$, and $r_{4}$). The combined equations used are as follows:

(2) $$X_{i}^{t+1}=\left\{\begin{array}{cc} X_{i}^{t}+r_{1}\cdot sin\left(r_{2}\right)\cdot \left|r_{3}\cdot X_{Best}^{t}-X_{i}^{t}\right| & \mathrm{if}\;r_{4}<0.5\\ X_{i}^{t}+r_{1}\cdot cos\left(r_{2}\right)\cdot \left|r_{3}\cdot X_{Best}^{t}-X_{i}^{t}\right| & \mathrm{if}\;r_{4}\geq 0.5 \end{array}\right.,$$

where $X_{i}^{t}$ is the position of the current solution in the *i*-th dimension at the *t*-th iteration, $X_{Best}^{t}$ shows the best individual’s position at the *t*-th iteration, and $r_{1}$, $r_{2}$, $r_{3}$, and $r_{4}$ are random parameters. SCA uses the latter parameters to avoid entrapment in suboptimal solutions and to balance the exploration and exploitation processes.

- $r_{1}$ is a linearly decreasing parameter and is calculated as follows: $r_{1}=a-t\frac{a}{T_{max}}$, where *a* is a constant, *t* is the current iteration, and $T_{max}$ represents the maximum iterations allowed.
  This parameter conditions the movement of the solution either towards the best solution ($r_{1}<1$) or away from the best solution ($r_{1}>1$). The above equation allows for the balance between exploration and exploitation.
- $r_{2}$ has values in the range [0,2π] and determines how big the movement of a solution is towards or away from the destination.
- $r_{3}$ has values in the range [0,2] and is used to assign a weight to the destination, reinforcing or inhibiting the impact of the destination point on the updating process of the other solutions.
- $r_{4}$, with values in the range [0,1], is a switch between the sine and cosine functions.

#### 2.3.2. Grey Wolf Optimizer

The grey wolf optimizer (GWO) was proposed by Mirjalili [4], this metaheuristic is inspired by both the hunting behavior and social hierarchy of the grey wolf. Within the pack there are four types of social hierarchy:

- Alpha (α): these are wolves that are at the top of the hierarchy and lead the pack.
- Beta (β): wolves that support the alpha wolves’ decisions.
- Delta (δ): they are strong but lack leadership skills.
- Omega: they have no power, they are dedicated to follow, help, and protect the younger members of the pack.

Applying the previously described hierarchy, at each step we will denote the best three solutions as alpha, beta, and delta, and the other solutions as omega. Basically, this means that the optimization process follows the flow of the position of the three best wolves in the hierarchy. In addition, the prey will be the optimal solution of the solution.

Most of the logic follows the equations:

(3) $$\begin{array}{cc} \vec{X}\left(t+1\right) & =\vec{X_{p}}\left(t\right)-\vec{A}\cdot \vec{D} \end{array}$$

(4) $$\begin{array}{cc} \vec{D} & =\;|\vec{C}\cdot \vec{X_{p}}\left(t\right)-\vec{X}\left(t\right)| \end{array}$$

where *t* denotes the current iteration, $\vec{A}$ and $\vec{C}$ are coefficient vectors, $\vec{X_{p}}$ is the position vector of the prey, and $\vec{X}$ is the position of the wolf. Finally, the symbol “·” represents a multiplication operator. Vectors $\vec{A}$ and $\vec{C}$ are equal to:

(5) $$\begin{array}{cc} \vec{A} & =2a\cdot \vec{r_{1}}-\vec{a} \end{array}$$

(6) $$\begin{array}{cc} \vec{C} & =2\vec{r_{2}} \end{array}$$

where components of $\vec{a}$ are linearly decreased from 2 to 0 through iterations and $\vec{r_{1}}$, $\vec{r_{2}}$ are random vectors with values from [0,1], calculated for each wolf at each iteration. Vector $\vec{A}$ controls the trade-off between exploration and exploitation, while $\vec{C}$ always adds some degree of randomness. This is necessary because our agents can become stuck in local optima and most of the metaheuristics have a way of avoiding this.

Since we do not know the real position of the optimal solution, $\vec{X_{p}}$ depends on the three best solutions and the formulas for updating each of the agents (wolves) are:

(7) $$\begin{array}{cc} \vec{D_{\alpha }} & =\;|\vec{C_{1}}\cdot {\vec{X}}_{\alpha }-\vec{X}| \end{array}$$

(8) $$\begin{array}{cc} \vec{D_{\beta }} & =\;|\vec{C_{2}}\cdot {\vec{X}}_{\beta }-\vec{X}| \end{array}$$

(9) $$\begin{array}{cc} \vec{D_{\delta }} & =\;|\vec{C_{3}}\cdot {\vec{X}}_{\delta }-\vec{X}| \end{array}$$

(10) $$\begin{array}{cc} {\vec{X}}_{1} & ={\vec{X}}_{\alpha }-{\vec{A}}_{1}\cdot \vec{D_{\alpha }} \end{array}$$

(11) $$\begin{array}{cc} {\vec{X}}_{2} & ={\vec{X}}_{\beta }-{\vec{A}}_{2}\cdot \vec{D_{\beta }} \end{array}$$

(12) $$\begin{array}{cc} {\vec{X}}_{3} & ={\vec{X}}_{\delta }-{\vec{A}}_{3}\cdot \vec{D_{\delta }} \end{array}$$

(13) $$\begin{array}{cc} \vec{X}\left(t+1\right) & =\frac{\vec{X_{1}}+\vec{X_{2}}+\vec{X_{3}}}{3} \end{array}$$

where $\vec{X}$ is the current position of the agent and $\vec{X}\left(t+1\right)$ is the updated one. The formula above indicates that the position of the wolf will be updated according to the best three wolves from the previous iteration. Notice that it will not be exactly equal to the average of the three best wolves because of the vector $\vec{C}$ which adds a small random shift. This makes sense because, from one side, we want our agents to be guided by the best individuals, but from the other side, we do not want to become stuck in local optima.

#### 2.3.3. Whale Optimization Algorithm

The whale optimization algorithm (WOA) is a metaheuristic that was proposed by Mirjalili and Lewis [5]. Like the GWO, the whale metaheuristic is inspired by a hierarchy and a particular way of hunting called bubble-net hunting. The metaheuristic has three main phases:

(1) Exploration phase: search for the prey.
(2) Encircling the prey.
(3) Exploitation phase: attacking the prey using a bubble-net method.

Based on the three main phases mentioned above, at each step of the exploration to search for the best solution (prey), the search agent (whale) is updated based on a random agent and not on the best. The mathematical model behind this logic is the following:

(14) $$\begin{array}{cc} \vec{X}\left(t+1\right) & ={\vec{X}}_{Rand}-\vec{A}\cdot \vec{D} \end{array}$$

(15) $$\begin{array}{cc} \vec{D} & =\;|\vec{C}\cdot {\vec{X}}_{Rand}-\vec{X}| \end{array}$$

where $\vec{A}$ and $\vec{C}$ are coefficient vectors, and ${\vec{X}}_{Rand}$ is a random position vector selected from the current population. Vectors $\vec{A}$ and $\vec{C}$ are equal to:

(16) $$\begin{array}{cc} \vec{A} & =2\vec{a}\cdot \vec{r}-\vec{a} \end{array}$$

(17) $$\begin{array}{cc} \vec{C} & =2\vec{r} \end{array}$$

where $\vec{r}$ is a random vector in the range [0,1] and $\vec{a}$ decreases linearly from 2 to 0 during the iterations. In addition, if ∣A∣>1, then the search agent is forced to move away from a reference whale.

Humpback whales encircle the prey during hunting. Then, they consider the current best candidate solution as the best solution and near the optimal one. In short, here is the model of encircling behavior that is used to update the position of the other whales towards the best search agent:

(18) $$\begin{array}{cc} \vec{X}\left(t+1\right) & =\vec{X^{\prime }}\left(t\right)-\vec{A}\cdot \vec{D} \end{array}$$

(19) $$\begin{array}{cc} \vec{D} & =\;|\vec{C}\cdot \vec{X^{\prime }}\left(t\right)-\vec{X}\left(t\right)| \end{array}$$

where *t* is the current iteration, $\vec{X^{\prime }}$ is the position of the best solution, $\vec{X}$ refers to the position vector of a solution, and finally, $\vec{A}$ and $\vec{C}$ are coefficient vectors, as shown in Equations (16) and (17).

The exploitation phase combines two approaches, shrinking the encircling mechanism and a spiral update of the position mechanism. In the shrinking encircling mechanism, the value of *A* is random within the interval [−a,a], and the value decreases from 2 to 0 as previously stated in Equation (16). In the spiral position update mechanism, we start by calculating the distance between the search agent (whale) and the best solution (prey), the mathematical model that simulates this movement is as follows:

(20) $$\begin{array}{cc} \vec{X}\left(t+1\right) & =\vec{D^{\prime }}\cdot e^{bl}\cdot \cos\left(2\pi l\right)+\vec{X^{\prime }} \end{array}$$

(21) $$\begin{array}{cc} \vec{D^{\prime }} & =|\vec{X^{\prime }}-\vec{X}t| \end{array}$$

where $\vec{D^{\prime }}$ represents the distance between the whale and the prey (best solution obtained so far), ***l*** is a random number between [−1,1] with a uniform distribution, and *b* is a constant defining the shape of the logarithmic spiral. Finally, the mathematical model that combines the two mechanisms is as follows:

(22) $$\begin{array}{cc} \; & \vec{X}\left(t+1\right)=\left\{\begin{array}{cc} \vec{X^{\prime }}\left(t\right)-\vec{A}\cdot \vec{D} & \mathrm{If}\;p<0.5\\ \vec{D^{\prime }}\cdot e^{bl}\cdot \cos\left(2\pi l\right)+\vec{X^{\prime }} & \mathrm{If}\;p\geq 0.5 \end{array}\right. \end{array}$$

where *p* is a random number in the range [0,1] and represents the probability of selecting one of these two methods to update the position of the whales.

### 2.4. Hybridization: Binarization Schemes Selector

In the literature, there are several related works on binarization [30,42] that have laid the groundwork for investigations into this domain problem, as there are several practical applications where working in binary domains is necessary. Moreover, research has emerged on how the change of binarization schemes affects each iteration of the search process, such as time-varying binarization schemes [38] or binarization scheme selectors [2,32,60], where the influence of binarization schemes and their impact at both the problem level and each iteration of the search has been demonstrated.

In Section 2.1, we can see how to adapt a continuous MH so that it can solve binary combinatorial problems. The two-step technique provides us with different possible combinations for binarizing continuous solutions. Although it is desirable to have a wide variety of combinations, each one must be tested individually to determine which is the best for a given problem.

In the literature, various related works have proposed the hybridization of the sine cosine algorithm, grey wolf optimizer, whale optimization algorithm, and Q-learning [2,31,36,40]. Q-learning was used as a dynamic binarization scheme selector in each of the metaheuristics, allowing them to solve binary combinatorial problems. This solution delegates the selection of a good binarization scheme to an artificial intelligence, eliminating any human bias.

The BSS provides a way to dynamically choose the binarization scheme, in this case the combination of transfer functions and binarization rules, within a set of them, using the scheme proposed in [2], where an intelligent operator chooses between a set of actions (possible binarization schemes) by observing the environment (exploration or exploitation).

#### 2.4.1. Actions

**Figure 4:**
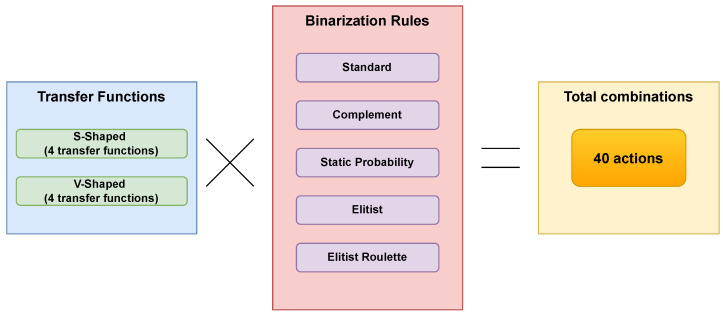
Building actions on the basis of binarization schemes.

The decision of which action to take is a complex task that requires careful evaluation of multiple options in different situations. In this context, Q-learning is utilized, which is a reinforcement learning technique that seeks to determine the best action to take in a specific state. In this work, as in previous studies, we define the considered actions as the existing combinations between the transfer functions (Table 1 and Table 2) and the binarization rules (Table 4). As an example, Figure 4 shows how the possible 40 actions that Q-learning would choose if it were working only with S- and V-type transfer functions and the five defined binarization rules, would be formed.

#### 2.4.2. States

As can be seen in Section 2.3, metaheuristics perform the search process alternating between intensification (exploration) and diversification (exploitation). Previous proposals [2,31,40,60,61] used these phases as the states to use in Q-learning. In these papers, the authors determined the stage of the search process by calculating the population diversity. In particular, they used the diversity proposed by Hussain Kashif et al. [62], which is defined as follows:

(23) $$Div=\frac{1}{l\cdot n}\sum_{d=1}^{l}\sum_{i=1}^{n}\left|{\bar{x}}^{d}-x_{i}^{d}\right|,$$

where Div represents the diversity status determination, ${\bar{x}}^{d}$ denotes the mean values of the individuals in dimension *d*, $x_{i}^{d}$ denotes the value of the *i*-th individual in dimension *d*, *n* denotes the population size, and *l* denotes the size of the individuals dimension.

If we consider the exploration and exploitation percentages to be XPL% (exploration) and XPT% (exploitation), the percentages XPL% and XPT% are computed from the study of Morales-Castañeda et al. [63] as follows:

(24) $$XPL\%=\frac{Div}{Div_{max}}\cdot 100,$$

(25) $$XPT\%=\frac{|Div-Div_{max}|}{Div_{max}}\cdot 100.$$

where Div represents the diversity state determined by Equation (23) and $Div_{max}$ denotes the maximum value of the diversity state discovered throughout the optimization process.

## 3. The Proposal: Analysis of Different Sets of Actions

Analyzing all the work presented so far and considering what is stated in Section 2.2, we ask ourselves the following questions:

(1) Which will have more impact on binarization, the transfer function or the binarization rule?
(2) Will the binarization schemes selector work better with more actions?

**Figure 5:**
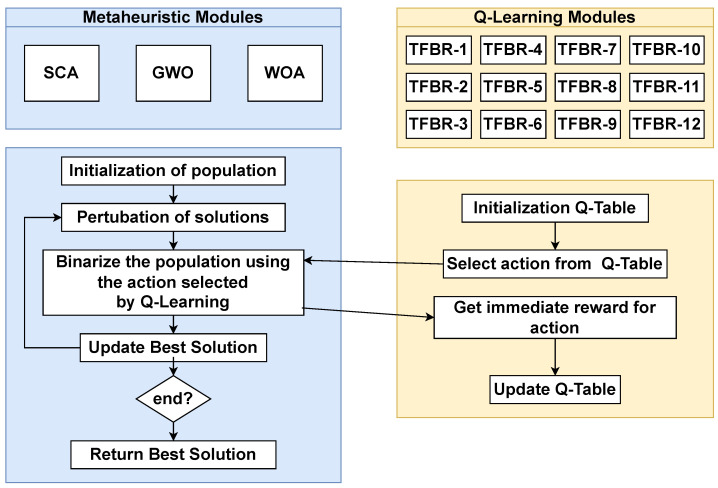
Proposal.

To answer these two questions, we apply the scheme proposed in Figure 5. For our analysis we used three continuous metaheuristics, which are SCA, GWO, and WOA. These three metaheuristics solved the set covering problem, a classical combinatorial optimization problem that will be defined in Section 4.1. Since it is a combinatorial problem and the chosen metaheuristics solve continuous problems, it is necessary to binarize the solutions.

As noted in Section 2.2, we have different ways of binarizing. On the other hand, Section 2.4 explains hybridizations where machine learning techniques are used to select binarization scheme dynamically.

We use the hybridization proposed in Section 2.4 as it allows us to evaluate how well a machine learning technique performs against different sets of actions.

In other words, we analyzed different combinations between four families of transfer functions (S-shaped, V-shaped, X-shaped, and Z-shaped) and the five binarization rules were carried out. Fourteen different action sets will be analyzed. First, a total of five sets with eight actions will be formed, where the S-shaped and V-shaped families will be used as transfer functions and the binarization function will be fixed in each set. Secondly, another five sets with 18 actions will be formed, where the S-shaped, V-shaped, X-shaped, and Z-shaped families will be used as transfer functions and the binarization function will be fixed in each set. Thirdly, a set with 40 actions will be formed, replicating the same work presented by the authors in [2,31,36,40]. Finally, a set with 80 actions will be formed, where the S-shaped, V-shaped, X-shaped, and Z-shaped families will be used as transfer functions and all the binarization functions will be used.

We present Table 5, which shows a set of actions analyzed in our study. The table includes 12 sets of actions, labeled as TFBR-1 to TFBR-12, and provides information on their transfer functions and binarization rules. The transfer functions considered are S-shaped, V-shaped, X-shaped, and Z-shaped, while the binarization rules used are standard, complement, static probability, elitist, and roulette elitist. The last column of the table reports the amount of actions associated with each set. The sets differ in terms of the combination of transfer functions and binarization rules used, and the number of actions considered. Table 5 serves as a reference for the subsequent experiments, in which we evaluate the performance of different algorithms on each set of actions.

The experimental results are presented in Section 4. In particular, in Section 4.2, the results obtained in each algorithm executed will be observed. Section 4.3 will analyze the convergence of each algorithm executed. In Section 4.4, we will analyze the exploration and exploitation behavior of each algorithm executed thanks to Equations (24) and (25). Finally, in Section 4.5, we will analyze the statistical test performed where all the executed versions are compared.

## 4. Experimental Results

To evaluate the performance of the proposed algorithms, the test cases of the set covering problem proposed in Beasley’s OR-Library [64] were used. In particular, 45 instances of this problem were solved. The algorithms were developed using the Python 3.7 programming language and executed using the free Google Colaboratory services [65]. The results obtained were stored and processed through databases provided by Google Cloud Platform.

Following recommendations from the literature [58], 40,000 calls were made to the objective function in each run. To achieve this, a population of 40 individuals and 1000 iterations were used across all GWO, SCA, and WOA runs. Thirty-one independent runs were performed for each instance. As for the parameters used for GWO, SCA, WOA, and Q-learning, they are detailed in Table 6.

### 4.1. Set Covering Problem

The set covering problem (SCP) is a classic NP-hard combinatorial optimization problem [66] and consists of finding the set of elements with the lowest cost that meets a certain amount of needs. The objective function of the problem is as follows:

(26) $$A=\left(\begin{array}{cccc} a_{11} & a_{12} & \cdots & a_{1n}\\ a_{21} & a_{22} & \cdots & a_{2n}\\ \cdots & \cdots & \cdots & \cdots \\ a_{m1} & a_{m2} & \cdots & a_{mn} \end{array}\right)$$

(27) $$Minimize\;Z=\sum_{j=1}^{n}c_{j}x_{j}$$

Subject to the following restrictions:

(28) $$\begin{array}{cc} \; & \sum_{j=1}^{n}a_{ij}x_{j}\geq 1\;\forall i\in I\\ \; & x_{j}\in \left\{0,1\right\}\;\forall j\in J \end{array}$$

where *A* is a binary matrix of size m rows and n columns and $a_{ij}\in \left\{0,1\right\}$ is the value of each cell in the matrix *A*. *i* and *j* are the sizes of the m rows and n columns. In the event that column *j* satisfies a row *i*, then $a_{ij}$ is equal to 1, otherwise it is 0. In addition, it has an associated cost c∈C, where $C=\{c_{1},c_{2},\cdots ,c_{n}\}$, together with i={1,2,⋯,m} and j={1,2,⋯,n}, which are the sets of rows and columns, respectively. Finally, *x* corresponds to the area to be covered.

The mathematical model of the set covering problem is explained in more detail in [67]. This problem formulation has inspired the modeling of different real-world problems such as airline and bus crew scheduling [68], the location of gas detectors for industrial plants [69], plant location selection [70], the location of emergency services [71], dynamic vehicle routing problems [72], the location of electric vehicle charging points in California [73], disaster management systems [74], emergency humanitarian logistics [75], the optimal UAV locations for the purpose of generating wireless communication networks in disaster areas [76], among others.

These studies allow us to appreciate the importance of solving this problem with optimization techniques that guarantee good results.

### 4.2. Summary of Results

This section will present an analysis of the results obtained using the three metaheuristics. Table 7, Table 8 and Table 9 display the relative percentage deviation (RPD) between the optimum and the best result obtained for 45 instances of the set covering problem using GWO, SCA, and WOA, respectively, with 12 different action sets. The RPD is defined in Equation (29), and the values of the RPD are grouped into four ranges. The first range corresponds to a deviation of 0%, the second range to a deviation of more than 0% up to 3%, the third range to a deviation of more than 3% up to 5%, and the last range corresponds to a deviation greater than 5%. The 12 different combinations are called TFBR-1 to TFBR-12, and the values shown in the table are the number of instances for each MH and evaluated combination that are within the RPD range indicated in each row.

The table presents thirteen columns, the first column indicates the four different RPD ranges and from column two to column thirteen indicates for each algorithm executed the number of instances whose RPD obtained is within the range.

(29) $$\mathrm{RPD}=\frac{100\cdot \left(Best-Opt\right)}{Opt}.$$

Table 10 and Table 11 show the results obtained with the twelve sets applied to the grey wolf optimizer (GWO). Table 12 and Table 13 show the results obtained with the twelve sets applied to the sine cosine algorithm (SCA). Table 14 and Table 15 show the results obtained with the twelve sets applied to the whale optimization algorithm (WOA).

For all tables, the first column indicates the name the OR-Library instances solved (Inst.), the second column the optimal value known for each of these instances (Opt.), the following three columns are repeated for each executed set. The first one indicates the best result obtained for the 31 independent runs, the second one indicates the average of the 31 results obtained, and the third one indicates the RPD.

#### 4.2.1. Analysis of the Results Obtained with Grey Wolf Optimizer

As can be seen in Table 7, only five sets achieved an RPD=0, i.e., reached the known optimum. In particular, TFBR-5 achieved this in 12 instances, TFBR-6 as well as TFBR-11 and TFBR-12 achieved this in 8 instances, and TFBR-10 achieved this in one instance.

For RPD∈]0,3], all sets achieved fitness in this range. In particular, TFBR-12 as well as TFBR-11 achieved this in 33 instances, TFBR-6 achieved this in 32 instances, TFBR-5 achieved this in 30 instances, TFBR-10 achieved this in 25 instances, TFBR-1 achieved this in 20 instances, TFBR-4 as well as TFBR-7 achieved this in 13 instances, TFBR-9 achieved this in 7 instances, and TFBR-2 as well as TFBR-3 and TFBR-8 achieved this in 1 instance.

For RPD∈]3,5], all polls achieved fitness in this range. In particular, TFBR-7 achieved this in 17 instances, TFBR-1 as well as TFBR-9 achieved this in 11 instances, TFBR-4 achieved this in 10 instances, TFBR-10 achieved this in 8 instances, TFBR-3 achieved this in 6 instances, TFBR-6 achieved this in 5 instances, TFBR-11 as well as TFBR-12 achieved this in 4 instances, TFBR-2 as well as TFBR-5 achieved this in 3 instances, and TFBR-8 achieved this in 2 instances.

Finally, for RPD>5, only eight sets achieved fitness in this range. In particular, TFBR-8 achieved this in 42 instances, TFBR-2 achieved this in 41 instances, TFBR-3 achieved this in 38 instances, TFBR-9 achieved this in 27 instances, TFBR-4 achieved this in 22 instances, TFBR-7 achieved this in 15 instances, TFBR-1 achieved this in 14 instances, and TFBR-10 achieved this in 11 instances.

Table 16 shows a ranking of the best sets of actions considering only the RPD obtained. From this table, the first and second best sets include the elitist binarization rule (TFBR-5: S-shaped and V-shaped × elitist and TFBR-11: S-shaped, V-shaped, X-shaped, and Z-shaped × elitist) and the third and fourth best sets include the roulette elitist binarization rule (TFBR-12: S-shaped, V-shaped, X-shaped, and Z-shaped × roulette elitist and TFBR-6: S-shaped and V-shaped × roulette elitist).

On the other hand, the worst and second worst sets include the standard binarization rule (TFBR-8: S-shaped, V-shaped, X-shaped, and Z-shaped × standard and TFBR-2: S-shaped and V-shaped × standard) and the third and fourth worst sets include the complement binarization rule (TFBR-3: S-shaped and V-shaped × complement and TFBR-9: S-shaped, V-shaped, X-shaped, and Z-shaped × complement).

In terms of the number of actions in each set, the best sets have 8 and 16 actions. In contrast, the sets with the most actions (TFBR-1 with 40 actions and TFBR-7 with 80 actions) are in the middle of the ranking.

Looking at these results, we can see that the binarization rule has a greater impact than the transfer functions. Moreover, increasing the number of actions does not imply better results.

#### 4.2.2. Analysis of the Results Obtained with Sine Cosine Algorithm

As can be seen in Table 8, only five sets achieved a RPD=0, i.e., reached the known optimum. In particular, TFBR-6 achieved this in seven instances, TFBR-5 as well as TFBR-11 and TFBR-12 achieved this in six instances, and TFBR-7 achieved this in one instance.

For RPD∈]0,3], only eight sets achieved fitness in this range. In particular, TFBR-12 as well as TFBR-5 and TFBR-6 achieved this in 33 instances, TFBR-11 achieved this in 31 instances, TFBR-7 as well as TFBR-9 achieved this in 14 instances, TFBR-1 achieved this in 13 instances, and TFBR-3 achieved this in 9 instances.

For RPD∈]3,5], only eight sets achieved fitness in this range. In particular, TFBR-7 achieved this in 21 instances, TFBR-1 achieved this in 16 instances, TFBR-9 achieved this in 10 instances, TFBR-11 achieved this in 7 instances, TFBR-5 as well as TFBR-12 achieved this in 5 instances, TFBR-6 achieved this in 4 instances and TFBR-3 achieved this in 1 instance.

Finally, for RPD>5, all sets achieved fitness in this range. In particular, TFBR-2 as well as TFBR-4, TFBR-8, and TFBR-10 achieved this in 45 instances, TFBR-3 achieved this in 44 instances, TFBR-9 achieved this in 21 instances, TFBR-1 as well as TFBR-1 achieved this in 16 instances, TFBR-5 as well as TFBR-6, TFBR-11, and TFBR-12 achieved this in 1 instance.

Table 17 shows a ranking of the best sets of actions considering only the RPD obtained. From this table, the best set includes the roulette elitist binarization rule (TFBR-6: S-shaped and V-shaped × roulette elitist), the second best set includes the elitist binarization rule (TFBR-5: S-shaped and V-shaped × elitist), the third best set also includes the roulette elitist binarization rule (TFBR-12: S-shaped, V-shaped, X-shaped, and Z-shaped × roulette elitist), and the fourth best set also includes the elitist binarization rule (TFBR-11: S-shaped, V-shaped, X-shaped, and Z-shaped × elitist).

On the other hand, the worst set includes the static probability binarization rule (TFBR-10: S-shaped, V-shaped, X-shaped, and Z-shaped x static probability), the second worst set includes the standard binarization rule (TFBR-8: S-shaped, V-shaped, X-shaped, and Z-shaped × standard), the third worst set also includes the static probability binarization rule (TFBR-4: S-shaped and V-shaped × static probability), and the fourth worst set also includes the standard binarization rule (TFBR-2: S-shaped and V-shaped × standard).

In terms of the number of actions in each set, the best sets have 8 and 16 actions. In contrast, the sets with the most actions (TFBR-1 with 40 actions and TFBR-7 with 80 actions) are in the middle of the ranking.

Looking at these results, we can see that the binarization rule has a greater impact than the transfer functions. Moreover, increasing the number of actions does not imply better results.

#### 4.2.3. Analysis of the Results Obtained with Whale Optimization Algorithm

As can be seen in Table 9, only six sets achieved an RPD=0, i.e., reached the known optimum. In particular, TFBR-6 achieved this in 10 instances, TFBR-5 achieved this in 8 instances, TFBR-12 achieved this in 7 instances, TFBR-11 achieved this in 6 instances, and TFBR-1 as well as TFBR-9 achieved this in 1 instance.

For RPD∈]0,3], only nine sets achieved fitness in this range. In particular, TFBR-5 achieved this in 32 instances, TFBR-11 achieved this in 31 instances, TFBR-6 achieved this in 29 instances, TFBR-12 achieved this in 27 instances, TFBR-7 achieved this in 22 instances, TFBR-9 achieved this in 21 instances, TFBR-1 achieved this in 18 instances, TFBR-2 achieved this in 4 instances, and TFBR-3 achieved this in 1 instance.

For RPD∈]3,5], only 10 sets achieved fitness in this range. In particular, TFBR-1 achieved this in 18 instances, TFBR-7 as well as TFBR-9 achieved this in 15 instances, TFBR-12 achieved this in 11 instances, TFBR-11 achieved this in 7 instances, TFBR-6 achieved this in 6 instances, TFBR-5 achieved this in 5 instances, TFBR-4 achieved this in 3 instances, and TFBR-2 as well as TFBR-3 achieved this in 1 instance.

Finally, for RPD>5, only nine sets achieved fitness in this range. In particular, TFBR-8 as well as TFBR-10 achieved this in 45 instances, TFBR-3 achieved this in 44 instances, TFBR-4 achieved this in 41 instances, TFBR-2 achieved this in 40 instances, TFBR-1 as well as TFBR-7 and TFBR-9 achieved this in 8 instancesm and TFBR-11 achieved this in 1 instance.

Table 18 shows a ranking of the best sets of actions considering only the RPD obtained. From this table, the best set includes the roulette elitist binarization rule (TFBR-6: S-shaped and V-shaped × roulette elitist), the second best set includes the elitist binarization rule (TFBR-5: S-shaped and V-shaped × elitist), the third best set also includes the roulette elitist binarization rule (TFBR-12: S-shaped, V-shaped, X-shaped, and Z-shaped × roulette elitist), and the fourth best set also includes the elitist binarization rule (TFBR-11: S-shaped, V-shaped, X-shaped, and Z-shaped × elitist).

On the other hand, the worst set includes the static probability binarization rule (TFBR-10: S-shaped, V-shaped, X-shaped, and Z-shaped x static probability), the second worst set includes the standard binarization rule (TFBR-8: S-shaped, V-shaped, X-shaped, and Z-shaped × standard), the third worst set includes the complement binarization rule (TFBR-3: S-shaped and V-shaped × complement), and the fourth worst set also includes the standard binarization rule (TFBR-2: S-shaped and V-shaped × standard).

In terms of the number of actions in each set, the best sets have 8 and 16 actions. In contrast, the sets with the most actions (TFBR-1 with 40 actions and TFBR-7 with 80 actions) are in the middle of the ranking.

Looking at these results, we can see that the binarization rule has a greater impact than the transfer functions. Moreover, increasing the number of actions does not imply better results.

### 4.3. Convergence Analysis

**Figure 6:**
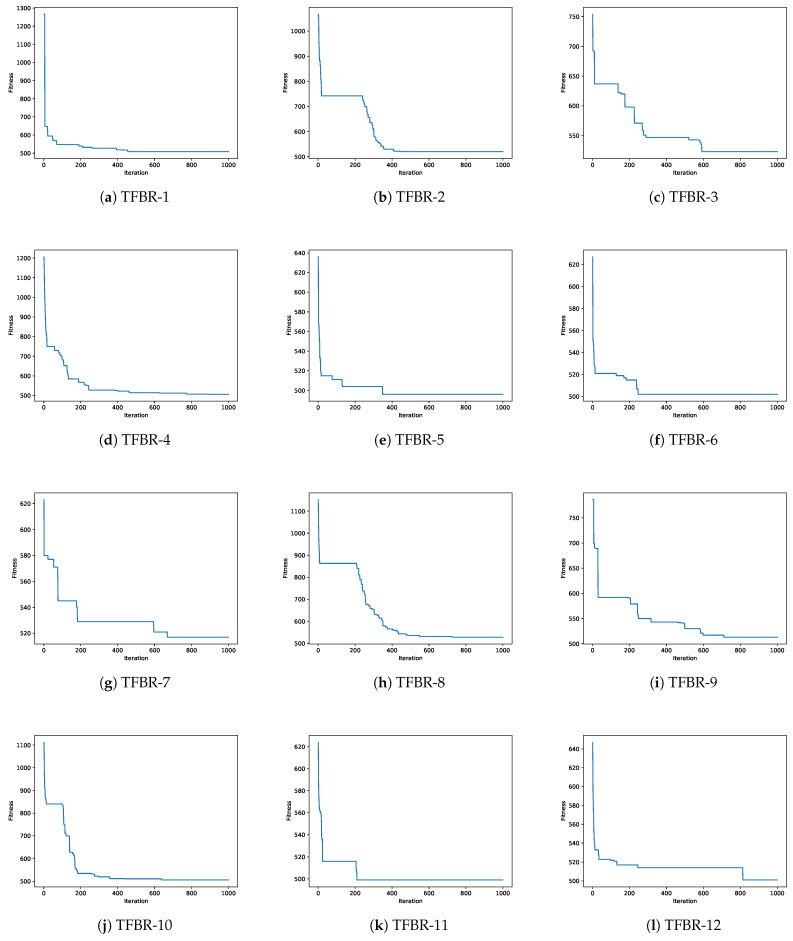
Convergence graphs of the best execution obtained for the scp44 instance using GWO.

**Figure 7:**
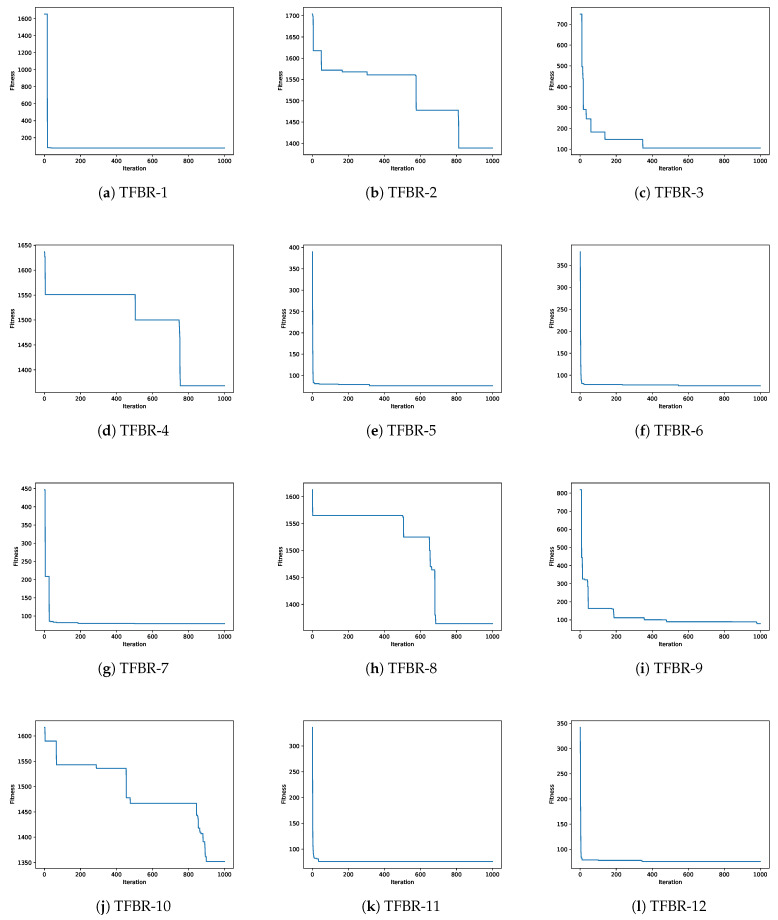
Convergence graphs of the best execution obtained for the scpb2 instance using SCA.

**Figure 8:**
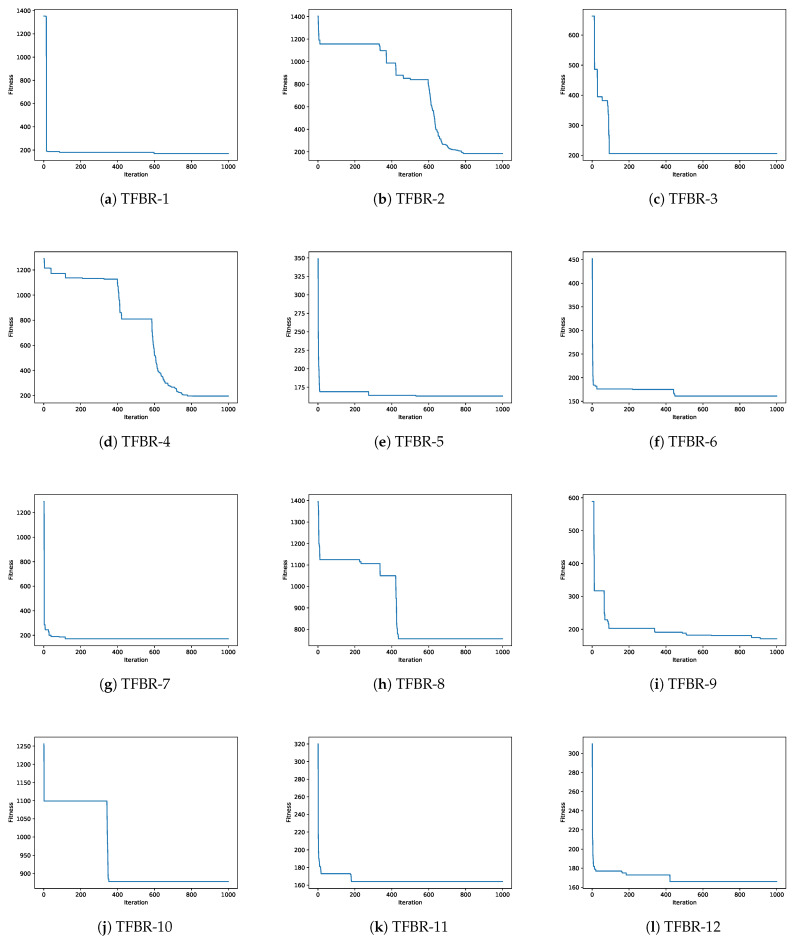
Convergence graphs of the best execution obtained for the scp65 instance using WOA.

In this section, a convergence analysis will be presented for each metaheuristic using the 12 sets of actions. Figure 6 shows the 12 convergence graphs for the best execution of the 31 performed using the grey wolf optimizer solving the scp44 instance; Figure 7 shows the 12 convergence graphs for the best execution of the 31 performed using the sine cosine algorithm solving the scpb2 instance; and Figure 8 shows the 12 convergence graphs for the best execution of the 31 performed using the whale optimization algorithm solving the scp65 instance.

For all figures, the x-axis shows the 1000 iterations run and the y-axis shows the best fitness obtained during the optimization process.

#### 4.3.1. Analysis of the Convergence Graphs Using Grey Wolf Optimizer

To analyze the convergence of the 12 sets applied to the GWO, the scp44 instance was used as an example. Table 19 shows the ranking of the algorithms ordered from the best fitness obtained to the worst fitness obtained for the scp44 instance. The global optimum for the scp44 instance is 494.

Analyzing Figure 6e,f,i,k, we can see that the algorithms had a fast convergence but were able to exit them and obtain better results. The sets of actions in these algorithms incorporate the elitist and elitist roulette binarization rules and they are the best-performing sets.

On the other hand, analyzing Figure 6b,c,h, we can see that the algorithms had slow convergence, indicating that they explored more of the search space. The sets of actions in these algorithms incorporate the standard and complement binarization rules and they are the worst-performing sets.

Finally, analyzing Figure 6a,g, we can see that the algorithms had a fast convergence, and from their behavior we can say that they fell into local optima since they did not improve much after convergence. The sets of actions in these algorithms incorporate all of the binarization rules.

With all of the above analysis we can visualize that the binarization rules have a greater impact than the transfer functions.

#### 4.3.2. Analysis of the Convergence Graphs Using Sine Cosine Algorithm

To analyze the convergence of the 12 sets applied to SCA, the scpb2 instance was used as an example. Table 20 shows the ranking of the algorithms ordered from the best fitness obtained to the worst fitness obtained for the scpb2 instance. The global optimum for the scpb2 instance is 76.

Analyzing Figure 7e,f,k,l, we can see that the algorithms had a fast convergence but were able to exit them and obtained better results. The sets of actions in these algorithms incorporate the elitist and elitist roulette binarization rules and they are the best-performing sets, moreover, the four sets reached the global optimum.

On the other hand, analyzing Figure 7b,d,h,j, we can see that the algorithms had slow convergence, indicating that they explored the search space. The sets of actions in these algorithms incorporate the standard and static probability binarization rules and they are the worst-performing sets, moreover, the four sets performed very poorly, reaching values above 1000.

Finally, analyzing Figure 7a,g, we can see that the algorithms had a fast convergence and from their behavior, we can say that they fell into local optima since they did not improve much after convergence. The sets of actions in these algorithms incorporate all of the binarization rules.

With all of the above analysis, we can visualize that the binarization rules have a greater impact than the transfer functions.

#### 4.3.3. Analysis of the Convergence Graphs Using Whale Optimization Algorithm

To analyze the convergence of the 12 sets applied to WOA, the scp65 instance was used as an example. Table 21 shows the ranking of the algorithms ordered from the best fitness obtained to the worst fitness obtained for the scp65 instance. The global optimum for the scp65 instance is 161.

Analyzing Figure 8e,f,k,l, we can see that the algorithms had a fast convergence but were able to exit them and obtained better results. The sets of actions in these algorithms incorporate the elitist and elitist roulette binarization rules and they are the best-performing sets, moreover, the TFBR-6 set reached the global optimum.

On the other hand, analyzing Figure 8c,d,h,j, we can see that the algorithms had slow convergence, indicating that they explored the search space. The sets of actions in these algorithms incorporate the static probability and complement binarization rules and they are the worst-performing sets.

Finally, analyzing Figure 8a,g, we can see that the algorithms had a fast convergence and from their behavior, we can say that they fell into local optimum since they did not improve much since convergence. The sets of actions in these algorithms incorporate all binarization rules.

With all the above analysis we can visualize that the binarization rules have a greater impact than the transfer functions.

### 4.4. Exploration and Exploitation Analysis

**Figure 9:**
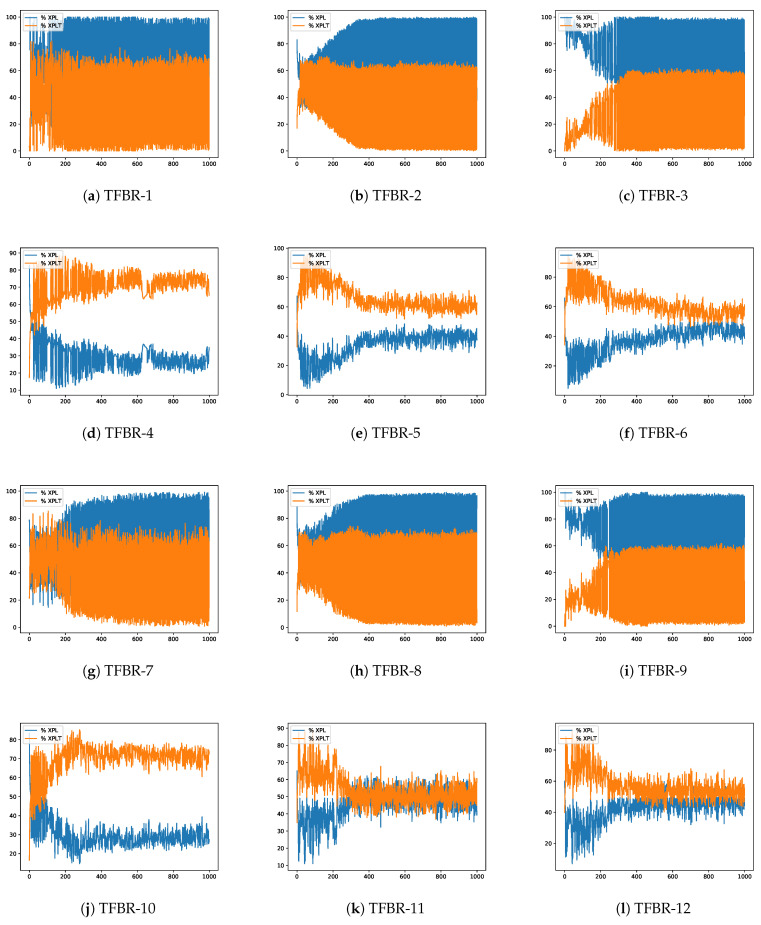
Exploration–exploitation graphs of the best execution obtained for the scp44 instance using GWO.

**Figure 10:**
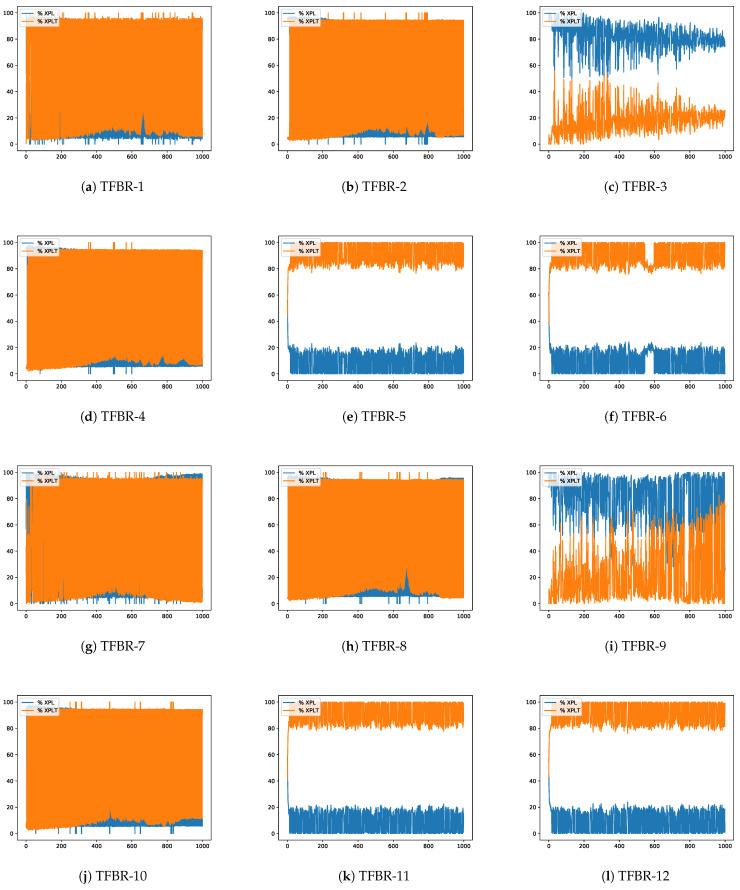
Exploration–exploitation graphs of the best execution obtained for the scpb2 instance using SCA.

**Figure 11:**
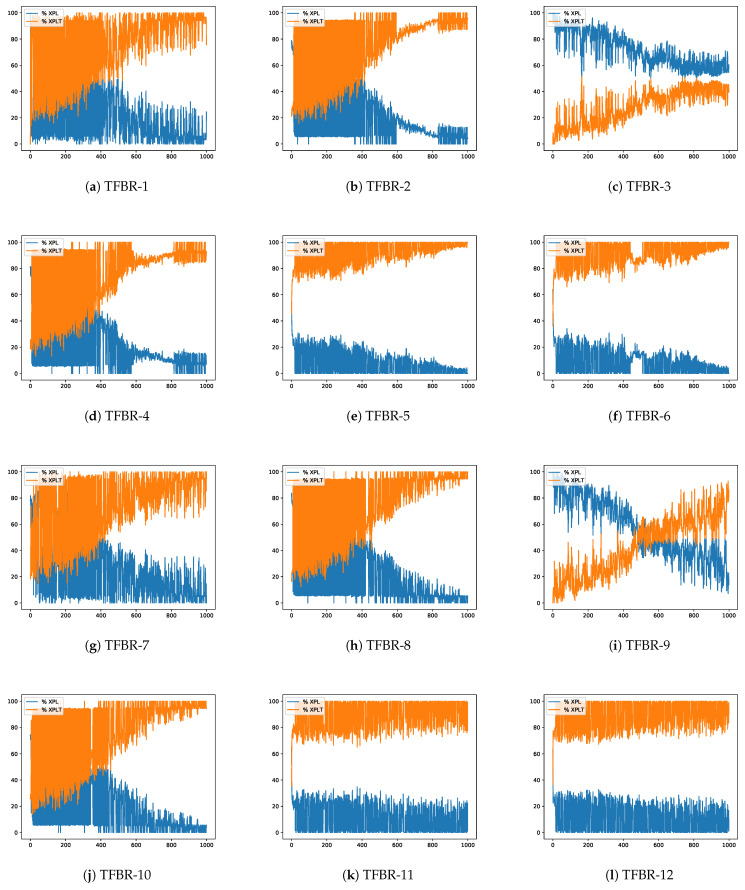
Exploration–exploitation graphs of the best execution obtained for the scp65 instance using WOA.

In Section 2.4.2, Equations (24) and (25) were presented. The first one indicates the exploration percentages of the search process and the second one indicates the exploitation percentage of the search process. This section will analyze the evolution of the exploration and exploitation percentages obtained in the best execution over the iterations in a given instance. The latter were randomly selected among the most representative of the experiments conducted as it is not possible to present all of them in this document. Nevertheless, all results and the implemented code have been made publicly available at https://github.com/joselemusr/BSS-the-Transfer-Function-really-important, (accessed on 10 August 2023). Figure 9 shows the 12 percentage graphs using the grey wolf optimizer solving the scp44 instance; Figure 10 shows the 12 percentage graphs using the sine cosine algorithm solving scpb2 instance; and Figure 11 shows the 12 percentage graphs using the whale optimization algorithm solving the scp65 instance.

For all figures, the x-axis shows the 1000 iterations run and the y-axis shows the percentage obtained during the optimization process. The exploration percentage is represented by the color blue and the exploitation percentage is represented by the color orange.

#### 4.4.1. Analysis of the Percentage Graphs Using Grey Wolf Optimizer

The instance selected to analyze the behavior of the the grey wolf optimizer is the same as the one selected in Section 4.3.1, i.e., the selected instance is the scp44.

Analyzing Figure 9e,f,k,l we can see that there is a good balance between exploration and exploitation, reaching percentages close to 50%. The set of actions in these algorithms incorporates the elitist and elitist roulette binarization rules. If we recall Table 19, these sets have the best performance.

On the other hand, analyzing Figure 9a–c,g–i we can see that there is a bad balance between exploration and exploitation. In particular, we can observe that the algorithms had high exploration rates throughout the iterations. The set of actions in these algorithms incorporates the elitist and elitist roulette binarization rules and these sets are the ones that presented the worst results.

Finally, analyzing Figure 9d,j we can see that they have high exploitation percentages. The set of actions in these algorithms incorporates the static probability binarization rule. If we consider the results obtained, we can conclude that both algorithms fell into local optima and did not have the ability to exit from it.

From the above, we can conclude that the binarization rules have a greater impact than the transfer functions.

#### 4.4.2. Analysis of the Percentage Graphs Using Sine Cosine Algorithm

The instance selected to analyze the behavior of the sine cosine algorithm is the same as the one selected in Section 4.3.2, i.e., the selected instance is the scpb2.

Analyzing Figure 10e,f,k,l we can see that all four algorithms show a high percentage of exploitation and that even only the first iteration shows some exploration. The set of actions in these algorithms incorporates the elitist and elitist roulette binarization rules. If we recall Table 20, these sets are the ones that had the best results, and they even reached the known global optimum.

On the other hand, analyzing Figure 10a,b,d,g,j we can see that the algorithms do not present a good balance between exploration and exploitation. In particular, they have a very aggressive behavior, where in one iteration they have a high percentage of exploration and in the next iteration they have a high percentage of exploitation. The set of actions in these algorithms incorporates the standard, static probability binarization rules and the algorithms incorporating all the binarization rules are present. Using the fitness values show in Table 20, we can see that aggressive behavior leads to good results in some cases and very good results in others.

Finally, analyzing Figure 10c,i we can see that they have high exploration percentages. Moreover, in most iterations, the algorithms show high exploration percentages. The set of actions in these algorithms incorporates the complement binarization rule. When reviewing Table 20 we can see that they reach acceptable results but when reviewing Table 12 and Table 13 we can see that they present a high average. This indicates that they have a stochastic behavior, providing low confidence.

In conclusion, we can see that the binarization rules have a greater impact than the transfer functions.

#### 4.4.3. Analysis of the Percentage Graphs Using Whale Optimization Algorithm

The instance selected to analyze the behavior of the whale optimization algorithm is the same as the one selected in Section 4.3.3, i.e., the selected instance is the scp65.

Analyzing Figure 11e,f,k,l we can see that all four algorithms show a high percentage of exploitation and that even only the first iteration shows some exploration. The set of actions in these algorithms incorporates the elitist and elitist roulette binarization rules. If we remember Table 21, these sets are the ones that had the best results, and one of them even reached the known global optimum.

On the other hand, analyzing Figure 11a,b,d,g,h,j we can see that the algorithms have an aggressive behavior at the beginning and as the iterations go by they present a more subdued behavior. The set of actions in these algorithms incorporates the standard, static probability binarization rules and the algorithms incorporating all the binarization rules are present. Considering the obtained results visible in Table 21, we can conclude that the algorithms fell into local optima and could not escape from there.

Finally, analyzing Figure 11c we can see that the algorithm presents a high exploration behavior that slowly decreases over the iterations. However, it does not reach high exploitation percentages. On the other hand, analyzing Figure 11i we can see that the algorithm has an explorative behavior that slowly decays until it reaches an exploitative behavior. The set of actions in these algorithms incorporates the complement binarization rule. Considering the obtained results visible in Table 21, we can conclude that they were in local optima and failed to intensify the search.

In conclusion, we can see that the binarization rules have a greater impact than the transfer functions.

### 4.5. Statistical Test

In order to determine the best action set, the Wilcoxon–Mann–Whitney test was applied. This is a non-parametric test [77] and it is used when the data are independent between samples and the data do not follow a normal distribution. Both characteristics are covered in this work since the data do not come from nature and each run performed was carried out independently of the others. As a sample size, 31 runs were used for each algorithm. The hypothesis used for this statistical test is the following:

$$H_{0}=\mathrm{Algorithm}\;A\geq \mathrm{Algorithm}\;B$$

$$H_{1}=\mathrm{Algorithm}\;A<\mathrm{Algorithm}\;B$$

If the result of the statistical test has obtained a *p*-value < 0.05, we cannot assume that Algorithm A has a worse performance than Algorithm B, rejecting $H_{0}$.

Table 22 shows the results when comparing the 12 sets of actions applied in the grey wolf optimizer; Table 23 shows the results when comparing the 12 action sets applied in the sine cosine algorithm; and Table 24 show the results when comparing the 12 action sets applied in the whale optimization algorithm. These tables are structured as follows: the first column presents the techniques used (Algorithm A); the following columns present the average *p*-values of the 45 instances compared with the version indicated in the column title (Algorithm B); if the value of this comparison is greater than 0.05 it is presented as “≥0.05”; when the comparison is against the same version the symbol “-” is presented; and the values have been approximated to the second decimal place

Analyzing the above three tables we can see that TFBR-5, TFBR-6, TFBR-11, and TFBR-12 were statistically better than TFBR-1, TFBR-2, TFBR-3, TFBR-4, TFBR-8, TFBR-9, and TFBR-10.

If we recall Table 5, the TFBR-5 and TFBR-11 sets incorporate the elitist binarization rule and their only difference is that the first set is made up of S-shaped and V-shaped transfer functions while the second set is made up of S-shaped, V-shaped, X-shaped, and Z-shaped transfer functions. On the other hand, the TFBR-6 and TFBR-12 sets incorporate the elitist roulette binarization rule and their only difference is that the first set is made up of the S-shaped and V-shaped transfer functions while the second set is made up of the S-shaped, V-shaped, X-shaped, and Z-shaped transfer functions.

These four sets are statistically better than the sets that incorporate the standard, complement, and static probability binarization rules. Moreover, they are better than those sets where all binarization rules are incorporated.

The fifth and sixth best sets are TFBR-1 and TFBR-7, which are those that include all the binarization rules and their only difference is that the first set is made up of the S-shaped and V-shaped transfer functions while the second set is made up of the S-shaped, V-shaped, X-shaped, and Z-shaped transfer functions.

With this in mind, we can observe that the best sets incorporate at least the elitist binarization rule or elitist roulette binarization rule. From this, we can conclude that the binarization rules have a greater impact than the transfer functions.

Table 25 shows the ranking of the sets by metaheuristic taking into account the statistical test applied. For each metaheuristic we have two columns, the first one refers to the name of the set and the second column refers to the number of times when the set was better than others. It is understood as a winning set when the comparison between them indicates a *p*-value lower than 0.05.

### 4.6. Summary of the Analysis

In Section 3, we presented two research questions which were as follows.

(1) Which will have more impact on binarization, the transfer function or the binarization rule?
(2) Will the binarization schemes selector work better with more actions?

If we analyze Table 25, we can see that for the three metaheuristics used in the research the best sets of actions are shown in Table 26.

As can be seen in Table 26, the best action sets are composed of the elitist binarization rule and the elitist roulette binarization rule. Therefore, we can conclude that the binarization rules have a greater impact than the transfer functions and that the intelligent binarization scheme selector does not perform better by incorporating more actions.

## 5. Conclusions and Outlook

Continuous metaheuristics are a class of evolutionary algorithms that are used to solve combinatorial problems. This makes them a powerful tool for solving binary problems, as they can efficiently explore a large number of possible solutions. However, it is necessary to incorporate intermediate steps to convert continuous solutions to a binary domain. These techniques are also capable of avoiding falling into local optima and finding high-quality solutions in problems with a large number of variables. This work has important implications in industry, as the set covering problem is a key problem in many applications, such as production, logistics, project planning, and resource allocation. Using MH optimization techniques to tackle this problem helps to improve efficiency and reduce costs. Additionally, the proposed approach in this work allows for selecting the best combination of binarization rules and transfer functions for a given problem instance, leading to even better performance.

A proposal is presented to improve the performance of metaheuristic optimization algorithms by using differentiated sets of actions. These sets of actions are composed of combinations of binarization rules and transfer functions. Twelve different sets of actions were proposed and applied to three different metaheuristic optimization algorithms: grey wolf optimizer, sine cosine algorithm, and whale optimization algorithm. These algorithms were applied to 45 different instances of the set covering problem.

The experimental results showed that the sets of actions that incorporate at least one elitist or elitist roulette binarization rule are the best, as they obtained the best results in terms of fitness and were statistically superior to the other sets of actions. Furthermore, it was found that binarization rules have a greater impact on the performance of metaheuristic algorithms than transfer functions. This work has demonstrated the importance of solving combinatorial binary problems using continuous metaheuristic techniques. Through the proposal that selects among a set of actions based on a reinforcement learning technique, it has been possible to improve the results obtained using traditional techniques. Additionally, it has been demonstrated that the elitist and elitist roulette binarization schemes are the most effective compared to the standard, complementary, and static probability schemes. This work opens a new line of research to improve the resolution of combinatorial binary problems using continuous metaheuristic techniques and their hybridization with machine learning techniques.

Conclusively, this study transcends conventional research on the binarization of continuous metaheuristics by not only providing a deeper understanding of this fundamental process but also by pioneering innovative approaches hitherto unexplored in the literature. Through a comprehensive and comparative analysis, we have tangibly illustrated how the judicious selection of transfer functions can make a substantial difference in the effectiveness and precision of binarization in the context of metaheuristics. These distinctively pinpoint contributions establish this study as a prominent reference in the research of this discipline, underscoring its significance in the optimization of continuous algorithms.

Future work could investigate how to use these optimization techniques on coverage problems with additional constraints, such as time or capacity constraints. It would also be interesting to investigate how these techniques behave with other algorithms, such as genetic algorithms and knowledge-based algorithms. Additionally, it would be important to investigate how these optimization techniques can be adapted to real-time coverage problems and how they can be integrated with existing automation systems in industry. In general, this work provides a solid foundation for future research in this area and demonstrates the importance of using optimization techniques in coverage problems.

## Figures and Tables

**Table 1:** S-shaped and V-shaped transfer functions.

Transfer Functions			
S-shaped [30,49]		V-shaped [30,49,50]	
Name	Equation	Name	Equation
S1	$T\left(d_{w}^{j}\right)=\frac{1}{1+e^{-2d_{w}^{j}}}$	V1	$T\left(d_{w}^{j}\right)=\left|erf\left(\frac{\sqrt{\pi }}{2}d_{w}^{j}\right)\right|$
S2	$T\left(d_{w}^{j}\right)=\frac{1}{1+e^{-d_{w}^{j}}}$	V2	$T\left(d_{w}^{j}\right)=\left|tanh(d_{w}^{j})\right|$
S3	$T\left(d_{w}^{j}\right)=\frac{1}{1+e^{\frac{-d_{w}^{j}}{2}}}$	V3	$T\left(d_{w}^{j}\right)=\left|\frac{d_{w}^{j}}{\sqrt{1+{\left(d_{w}^{j}\right)}^{2}}}\right|$
S4	$T\left(d_{w}^{j}\right)=\frac{1}{1+e^{\frac{-d_{w}^{j}}{3}}}$	V4	$T\left(d_{w}^{j}\right)=\left|\frac{2}{\pi }arctan\left(\frac{\pi }{2}d_{w}^{j}\right)\right|$

**Table 2:** X-shaped and Z-shaped transfer functions.

Transfer Functions			
X-shaped [51,52]		Z-shaped [53,54]	
Name	Equation	Name	Equation
X1	$T\left(d_{w}^{j}\right)=\frac{1}{1+e^{2d_{w}^{j}}}$	Z1	$T\left(d_{w}^{j}\right)=\sqrt{1-2^{d_{w}^{j}}}$
X2	$T\left(d_{w}^{j}\right)=\frac{1}{1+e^{d_{w}^{j}}}$	Z2	$T\left(d_{w}^{j}\right)=\sqrt{1-5^{d_{w}^{j}}}$
X3	$T\left(d_{w}^{j}\right)=\frac{1}{1+e^{\frac{d_{w}^{j}}{2}}}$	Z3	$T\left(d_{w}^{j}\right)=\sqrt{1-8^{d_{w}^{j}}}$
X4	$T\left(d_{w}^{j}\right)=\frac{1}{1+e^{\frac{d_{w}^{j}}{3}}}$	Z4	$T\left(d_{w}^{j}\right)=\sqrt{1-20^{d_{w}^{j}}}$

**Table 3:** X-shaped and Z-shaped transfer functions.

Time-Varying Transfer Functions [38]			
S-shaped		V-shaped	
Name	Equation	Name	Equation
$TV_{S1}$	$T\left(d_{w}^{j},\tau \right)=\frac{1}{1+e^{\frac{-2d_{w}^{j}}{\tau }}}$	$TV_{V1}$	$T\left(d_{w}^{j},\tau \right)=\left\{\begin{array}{cc} 1-\frac{2}{1+e^{\frac{-2d_{w}^{j}}{\tau }}} & d_{w}^{j}\leq 0\\ \frac{2}{1+e^{\frac{-2d_{w}^{j}}{\tau }}}-1 & d_{w}^{j}>0 \end{array}\right.$
$TV_{S2}$	$T\left(d_{w}^{j},\tau \right)=\frac{1}{1+e^{\frac{-d_{w}^{j}}{\tau }}}$	$TV_{V2}$	$T\left(d_{w}^{j},\tau \right)=\left\{\begin{array}{cc} 1-\frac{2}{1+e^{\frac{-d_{w}^{j}}{\tau }}} & d_{w}^{j}\leq 0\\ \frac{2}{1+e^{\frac{-d_{w}^{j}}{\tau }}}-1 & d_{w}^{j}>0 \end{array}\right.$
$TV_{S3}$	$T\left(d_{w}^{j},\tau \right)=\frac{1}{1+e^{\frac{-d_{w}^{j}}{2\tau }}}$	$TV_{V3}$	$T\left(d_{w}^{j},\tau \right)=\left\{\begin{array}{cc} 1-\frac{2}{1+e^{\frac{-d_{w}^{j}}{2\tau }}} & d_{w}^{j}\leq 0\\ \frac{2}{1+e^{\frac{-d_{w}^{j}}{2\tau }}}-1 & d_{w}^{j}>0 \end{array}\right.$
$TV_{S4}$	$T\left(d_{w}^{j},\tau \right)=\frac{1}{1+e^{\frac{-d_{w}^{j}}{3\tau }}}$	$TV_{V4}$	$T\left(d_{w}^{j},\tau \right)=\left\{\begin{array}{cc} 1-\frac{2}{1+e^{\frac{-d_{w}^{j}}{3\tau }}} & d_{w}^{j}\leq 0\\ \frac{2}{1+e^{\frac{-d_{w}^{j}}{3\tau }}}-1 & d_{w}^{j}>0 \end{array}\right.$

**Table 4:** Binarization rules.

Type	Binarization Rules
Standard	$X_{new}^{j}=\left\{\begin{array}{cc} 1 & if\;rand\leq T(d_{w}^{j})\\ 0 & else. \end{array}\right.$
Complement	$X_{new}^{j}=\left\{\begin{array}{cc} Complement(X_{w}^{j}) & if\;rand\leq T(d_{w}^{j})\\ 0 & else. \end{array}\right.$
Static Probability	$X_{new}^{j}=\left\{\begin{array}{cc} 0 & if\;T(d_{w}^{j})\leq \alpha \\ X_{w}^{j} & if\;\alpha <T\left(d_{w}^{j}\right)\leq \frac{1}{2}\left(1+\alpha \right)\\ 1 & if\;T\left(d_{w}^{j}\right)\geq \frac{1}{2}\left(1+\alpha \right) \end{array}\right.$
Elitist	$X_{new}^{j}=\left\{\begin{array}{cc} X_{Best}^{j} & if\;rand<T(d_{w}^{j})\\ 0 & else. \end{array}\right.$
Roulette Elitist	$X_{new}^{j}=\left\{\begin{array}{cc} P\left[X_{new}^{j}=\zeta_{j}\right]=\frac{f(\zeta )}{\sum_{\delta \in Q_{g}}f\left(\delta \right)} & \mathrm{if}\;\mathrm{rand}\;\leq T(d_{w}^{j})\\ P[X_{new}^{j}=0]=1 & else. \end{array}\right.$

**Table 5:** Set of actions analyzed.

Set of Actions			
Set ID	Transfer Functions	Binarization Rules	Amount of actions
TFBR-1	S-shaped and V-shaped	Standard, Complement, Static Probability, Elitist, and Roulette Elitist	40
TFBR-2	S-shaped and V-shaped	Standard	8
TFBR-3	S-shaped and V-shaped	Complement	8
TFBR-4	S-shaped and V-shaped	Static Probability	8
TFBR-5	S-shaped and V-shaped	Elitist	8
TFBR-6	S-shaped and V-shaped	Roulette Elitist	8
TFBR-7	S-shaped, V-shaped, X-shaped and Z-shaped	Standard, Complement, Static Probability, Elitist, and Roulette Elitist	80
TFBR-8	S-shaped, V-shaped, X-shaped, and Z-shaped	Standard	16
TFBR-9	S-shaped, V-shaped, X-shaped, and Z-shaped	Complement	16
TFBR-10	S-shaped, V-shaped, X-shaped, and Z-shaped	Static Probability	16
TFBR-11	S-shaped, V-shaped, X-shaped, and Z-shaped	Elitist	16
TFBR-12	S-shaped, V-shaped, X-shaped, and Z-shaped	Roulette Elitist	16

**Table 6:** Parameter settings.

Parameter	Value
Independent runs	31
Number of populations	40
Number of iterations	1000
Parameter *a* of SCA	2
Parameter *a* of GWO	Decreases linearly from 2 to 0
Parameter *a* of WOA	Decreases linearly from 2 to 0
Parameter *b* of WOA	1
Parameter α of Q-learning	0.1
Parameter γ of Q-learning	0.4

**Table 7:** RPD obtained for 12 sets using GWO.

RPD	TFBR-1	TFBR-2	TFBR-3	TFBR-4	TFBR-5	TFBR-6	TFBR-7	TFBR-8	TFBR-9	TFBR-10	TFBR-11	TFBR-12
0	0	0	0	0	12	8	0	0	0	1	8	8
]0,3]	20	1	1	13	30	32	13	1	7	25	33	33
]3,5]	11	3	6	10	3	5	17	2	11	8	4	4
>5	14	41	38	22	0	0	15	42	27	11	0	0

**Table 8:** RPD obtained for the 12 sets using SCA.

RPD	TFBR-1	TFBR-2	TFBR-3	TFBR-4	TFBR-5	TFBR-6	TFBR-7	TFBR-8	TFBR-9	TFBR-10	TFBR-11	TFBR-12
0	0	0	0	0	6	7	1	0	0	0	6	6
]0,3]	13	0	9	0	33	33	14	0	14	0	31	33
]3,5]	16	0	1	0	5	4	21	0	10	0	7	5
>5	16	45	44	45	1	1	9	45	21	45	1	1

**Table 9:** RPD obtained for 12 sets using WOA.

RPD	TFBR-1	TFBR-2	TFBR-3	TFBR-4	TFBR-5	TFBR-6	TFBR-7	TFBR-8	TFBR-9	TFBR-10	TFBR-11	TFBR-12
0	1	0	0	0	8	10	0	0	1	0	6	7
]0,3]	18	4	0	1	32	29	22	0	21	0	31	27
]3,5]	18	1	1	3	5	6	15	0	15	0	7	11
>5	8	40	44	41	0	0	8	45	8	45	1	0

**Table 10:** Results obtained with GWO and the TFBR-1, TFBR-2, TFBR-3, TFBR-4, TFBR-5, and TFBR-6 sets.

SCP		TFBR-1			TFBR-2			TFBR-3			TFBR-4			TFBR-5			TFBR-6		
**Inst.**	**Opt.**	**Best**	**Avg**	**RPD**	**Best**	**Avg**	**RPD**	**Best**	**Avg**	**RPD**	**Best**	**Avg**	**RPD**	**Best**	**Avg**	**RPD**	**Best**	**Avg**	**RPD**
41	429	431.0	436.81	0.47	439.0	472.35	2.33	435.0	448.48	1.4	432.0	445.48	0.7	430.0	433.23	0.23	430.0	434.29	0.23
42	512	532.0	543.48	3.91	562.0	611.42	9.77	551.0	578.97	7.62	543.0	558.97	6.05	521.0	530.32	1.76	518.0	529.39	1.17
43	516	535.0	542.71	3.68	567.0	626.97	9.88	543.0	577.71	5.23	532.0	551.94	3.1	517.0	525.68	0.19	522.0	526.1	1.16
44	494	508.0	521.29	2.83	520.0	581.87	5.26	523.0	539.68	5.87	506.0	530.13	2.43	496.0	507.39	0.4	502.0	509.29	1.62
45	512	532.0	545.23	3.91	547.0	602.03	6.84	552.0	581.77	7.81	529.0	552.0	3.32	520.0	526.42	1.56	521.0	527.29	1.76
46	560	576.0	582.39	2.86	592.0	670.77	5.71	582.0	617.52	3.93	574.0	591.94	2.5	566.0	570.26	1.07	563.0	570.29	0.54
47	430	437.0	444.19	1.63	454.0	492.52	5.58	449.0	468.06	4.42	441.0	451.81	2.56	434.0	436.87	0.93	433.0	436.9	0.7
48	492	502.0	508.45	2.03	510.0	598.45	3.66	522.0	547.52	6.1	506.0	524.29	2.85	494.0	500.45	0.41	493.0	500.29	0.2
49	641	670.0	688.58	4.52	684.0	763.71	6.71	679.0	727.52	5.93	685.0	705.81	6.86	652.0	669.13	1.72	661.0	671.68	3.12
410	514	524.0	529.71	1.95	552.0	601.65	7.39	541.0	557.55	5.25	522.0	545.68	1.56	518.0	522.48	0.78	519.0	523.13	0.97
51	253	261.0	266.87	3.16	277.0	305.16	9.49	264.0	283.9	4.35	263.0	272.87	3.95	257.0	261.19	1.58	255.0	262.55	0.79
52	302	324.0	331.48	7.28	349.0	381.45	15.56	332.0	353.74	9.93	333.0	341.32	10.26	315.0	322.9	4.3	316.0	322.45	4.64
53	226	231.0	234.06	2.21	245.0	270.29	8.41	236.0	247.52	4.42	233.0	239.81	3.1	228.0	230.23	0.88	229.0	230.94	1.33
54	242	249.0	252.52	2.89	254.0	283.29	4.96	255.0	265.77	5.37	252.0	260.06	4.13	244.0	248.0	0.83	245.0	248.29	1.24
55	211	215.0	218.26	1.9	221.0	245.84	4.74	220.0	228.55	4.27	216.0	224.74	2.37	212.0	214.55	0.47	212.0	215.48	0.47
56	213	218.0	227.45	2.35	240.0	267.87	12.68	221.0	243.68	3.76	224.0	233.71	5.16	214.0	219.06	0.47	215.0	220.52	0.94
57	293	307.0	312.03	4.78	320.0	354.61	9.22	317.0	333.13	8.19	308.0	318.97	5.12	298.0	303.03	1.71	297.0	303.0	1.37
58	288	294.0	298.42	2.08	314.0	338.06	9.03	303.0	319.06	5.21	295.0	308.77	2.43	290.0	293.9	0.69	291.0	294.52	1.04
59	279	285.0	289.68	2.15	301.0	330.13	7.89	293.0	307.87	5.02	287.0	297.84	2.87	281.0	284.55	0.72	280.0	284.03	0.36
510	265	274.0	278.42	3.4	292.0	318.77	10.19	284.0	296.42	7.17	274.0	283.87	3.4	268.0	272.06	1.13	266.0	271.16	0.38
61	138	144.0	146.58	4.35	155.0	219.35	12.32	150.0	169.94	8.7	146.0	150.87	5.8	141.0	143.23	2.17	140.0	143.06	1.45
62	146	153.0	156.39	4.79	171.0	264.4	17.12	166.0	199.29	13.7	151.0	160.61	3.42	148.0	150.0	1.37	146.0	150.58	0.0
63	145	147.0	150.32	1.38	189.0	250.65	30.34	152.0	184.58	4.83	149.0	155.74	2.76	145.0	148.06	0.0	147.0	148.42	1.38
64	131	133.0	135.0	1.53	147.0	199.0	12.21	138.0	151.16	5.34	134.0	138.45	2.29	131.0	132.77	0.0	131.0	132.9	0.0
65	161	173.0	178.61	7.45	188.0	271.29	16.77	188.0	215.16	16.77	173.0	180.42	7.45	161.0	168.35	0.0	162.0	169.0	0.62
a1	253	262.0	268.1	3.56	309.0	362.26	22.13	286.0	314.61	13.04	270.0	278.74	6.72	258.0	262.32	1.98	259.0	262.52	2.37
a2	252	266.0	271.81	5.56	316.0	364.42	25.4	285.0	314.1	13.1	271.0	280.77	7.54	258.0	263.61	2.38	259.0	265.29	2.78
a3	232	245.0	248.03	5.6	280.0	326.13	20.69	264.0	286.65	13.79	248.0	255.06	6.9	239.0	242.81	3.02	240.0	243.68	3.45
a4	234	247.0	251.26	5.56	273.0	332.42	16.67	269.0	295.65	14.96	247.0	262.19	5.56	236.0	242.52	0.85	236.0	242.87	0.85
a5	236	245.0	249.77	3.81	265.0	330.97	12.29	265.0	294.39	12.29	247.0	259.87	4.66	239.0	243.42	1.27	240.0	243.61	1.69
b1	69	71.0	72.58	2.9	137.0	211.19	98.55	106.0	132.29	53.62	71.0	76.94	2.9	69.0	70.29	0.0	69.0	70.06	0.0
b2	76	78.0	80.97	2.63	146.0	196.16	92.11	112.0	142.26	47.37	81.0	86.55	6.58	76.0	77.32	0.0	76.0	77.48	0.0
b3	80	82.0	84.13	2.5	161.0	240.94	101.25	129.0	166.84	61.25	84.0	90.13	5.0	80.0	81.06	0.0	81.0	81.45	1.25
b4	79	83.0	85.29	5.06	158.0	214.74	100.0	114.0	153.9	44.3	84.0	89.58	6.33	79.0	81.26	0.0	80.0	81.61	1.27
b5	72	73.0	75.0	1.39	140.0	193.06	94.44	101.0	137.39	40.28	73.0	82.68	1.39	72.0	72.87	0.0	72.0	72.81	0.0
c1	227	245.0	250.68	7.93	289.0	371.32	27.31	279.0	316.45	22.91	248.0	260.52	9.25	231.0	238.45	1.76	233.0	239.1	2.64
c2	219	232.0	241.48	5.94	295.0	364.77	34.7	268.0	305.77	22.37	239.0	252.68	9.13	226.0	230.23	3.2	227.0	231.32	3.65
c3	243	256.0	263.97	5.35	348.0	415.35	43.21	293.0	343.03	20.58	267.0	280.74	9.88	248.0	252.65	2.06	248.0	253.65	2.06
c4	219	232.0	237.06	5.94	314.0	359.52	43.38	270.0	303.9	23.29	233.0	247.97	6.39	225.0	230.9	2.74	226.0	230.52	3.2
c5	215	229.0	233.35	6.51	293.0	358.71	36.28	270.0	303.42	25.58	228.0	243.84	6.05	220.0	224.16	2.33	220.0	224.16	2.33
d1	60	65.0	66.94	8.33	171.0	250.42	185.0	130.0	174.23	116.67	66.0	72.77	10.0	60.0	61.61	0.0	61.0	62.03	1.67
d2	66	67.0	70.9	1.52	222.0	305.71	236.36	136.0	176.35	106.06	70.0	81.03	6.06	66.0	67.45	0.0	66.0	67.87	0.0
d3	72	77.0	79.97	6.94	230.0	314.58	219.44	159.0	209.1	120.83	82.0	90.35	13.89	73.0	74.71	1.39	73.0	75.23	1.39
d4	62	63.0	65.35	1.61	155.0	254.77	150.0	125.0	163.23	101.61	65.0	75.32	4.84	62.0	62.9	0.0	62.0	62.97	0.0
d5	61	65.0	66.74	6.56	159.0	250.29	160.66	139.0	170.06	127.87	66.0	75.13	8.2	61.0	62.35	0.0	61.0	62.68	0.0
		263.07	268.5	3.88	305.58	363.1	43.64	286.58	314.4	25.83	265.51	277.09	5.19	256.87	261.27	1.07	257.4	261.7	1.29

**Table 11:** Continued results obtained with GWO and the TFBR-7, TFBR-8, TFBR-9, TFBR-10, TFBR-11, and TFBR-12 sets.

SCP		TFBR-7			TFBR-8			TFBR-9			TFBR-10			TFBR-11			TFBR-12		
**Inst.**	**Opt.**	**Best**	**Avg**	**RPD**	**Best**	**Avg**	**RPD**	**Best**	**Avg**	**RPD**	**Best**	**Avg**	**RPD**	**Best**	**Avg**	**RPD**	**Best**	**Avg**	**RPD**
41	429	433.0	437.87	0.93	438.0	460.39	2.1	432.0	442.55	0.7	430.0	436.06	0.23	431.0	433.39	0.47	430.0	433.52	0.23
42	512	530.0	545.26	3.52	544.0	590.26	6.25	538.0	557.19	5.08	521.0	546.65	1.76	518.0	530.77	1.17	524.0	533.03	2.34
43	516	533.0	544.26	3.29	554.0	598.42	7.36	537.0	558.68	4.07	530.0	541.0	2.71	519.0	527.03	0.58	523.0	528.39	1.36
44	494	517.0	523.1	4.66	528.0	558.65	6.88	513.0	532.97	3.85	506.0	523.97	2.43	499.0	509.29	1.01	501.0	509.16	1.42
45	512	529.0	544.03	3.32	552.0	602.97	7.81	532.0	560.1	3.91	527.0	541.74	2.93	520.0	526.0	1.56	516.0	527.65	0.78
46	560	572.0	583.35	2.14	592.0	634.32	5.71	572.0	599.16	2.14	568.0	582.0	1.43	562.0	568.06	0.36	565.0	569.74	0.89
47	430	438.0	444.45	1.86	453.0	481.87	5.35	440.0	451.87	2.33	435.0	445.48	1.16	433.0	436.48	0.7	435.0	437.58	1.16
48	492	502.0	511.1	2.03	508.0	576.35	3.25	506.0	525.71	2.85	500.0	508.74	1.63	497.0	499.35	1.02	496.0	499.87	0.81
49	641	671.0	688.52	4.68	693.0	750.13	8.11	680.0	705.52	6.08	673.0	688.71	4.99	658.0	670.1	2.65	661.0	672.48	3.12
410	514	526.0	530.97	2.33	542.0	576.9	5.45	526.0	540.58	2.33	522.0	532.84	1.56	517.0	520.84	0.58	518.0	522.1	0.78
51	253	256.0	268.42	1.19	265.0	291.84	4.74	262.0	273.81	3.56	260.0	268.42	2.77	256.0	262.94	1.19	256.0	262.06	1.19
52	302	327.0	332.81	8.28	341.0	366.0	12.91	337.0	343.97	11.59	324.0	333.32	7.28	315.0	322.58	4.3	316.0	324.13	4.64
53	226	231.0	234.52	2.21	243.0	262.03	7.52	235.0	241.45	3.98	230.0	235.74	1.77	228.0	229.87	0.88	228.0	230.48	0.88
54	242	249.0	253.1	2.89	256.0	277.94	5.79	249.0	256.87	2.89	249.0	252.77	2.89	246.0	248.48	1.65	245.0	247.71	1.24
55	211	216.0	218.48	2.37	222.0	237.58	5.21	216.0	222.06	2.37	214.0	218.0	1.42	211.0	214.06	0.0	212.0	214.97	0.47
56	213	221.0	228.71	3.76	226.0	255.19	6.1	226.0	235.13	6.1	222.0	228.23	4.23	216.0	220.19	1.41	215.0	219.71	0.94
57	293	309.0	313.19	5.46	310.0	339.29	5.8	307.0	320.06	4.78	300.0	311.35	2.39	296.0	302.97	1.02	297.0	303.42	1.37
58	288	297.0	299.52	3.12	304.0	329.84	5.56	298.0	309.97	3.47	292.0	299.58	1.39	290.0	293.06	0.69	290.0	292.87	0.69
59	279	285.0	290.35	2.15	293.0	320.65	5.02	283.0	297.9	1.43	284.0	291.1	1.79	281.0	283.35	0.72	280.0	282.94	0.36
510	265	272.0	278.87	2.64	284.0	305.06	7.17	275.0	286.77	3.77	271.0	279.45	2.26	267.0	271.39	0.75	267.0	271.58	0.75
61	138	145.0	147.65	5.07	151.0	186.61	9.42	147.0	153.65	6.52	144.0	147.48	4.35	141.0	142.55	2.17	141.0	142.77	2.17
62	146	153.0	157.13	4.79	173.0	230.39	18.49	156.0	168.77	6.85	149.0	158.42	2.05	149.0	150.77	2.05	147.0	150.71	0.68
63	145	149.0	151.03	2.76	155.0	208.71	6.9	151.0	161.71	4.14	148.0	152.03	2.07	146.0	148.06	0.69	147.0	148.16	1.38
64	131	133.0	135.23	1.53	138.0	169.39	5.34	135.0	139.42	3.05	131.0	134.87	0.0	131.0	132.65	0.0	131.0	132.55	0.0
65	161	174.0	178.42	8.07	193.0	240.65	19.88	181.0	191.45	12.42	165.0	178.87	2.48	164.0	167.65	1.86	162.0	169.48	0.62
a1	253	262.0	268.52	3.56	284.0	337.74	12.25	274.0	286.97	8.3	264.0	271.74	4.35	259.0	261.84	2.37	258.0	261.87	1.98
a2	252	264.0	273.68	4.76	286.0	340.32	13.49	279.0	295.1	10.71	266.0	274.65	5.56	258.0	263.74	2.38	258.0	263.32	2.38
a3	232	246.0	249.39	6.03	277.0	307.9	19.4	254.0	265.06	9.48	240.0	250.58	3.45	238.0	242.0	2.59	237.0	242.35	2.16
a4	234	246.0	253.1	5.13	280.0	310.1	19.66	259.0	269.71	10.68	243.0	255.55	3.85	238.0	242.45	1.71	240.0	242.68	2.56
a5	236	248.0	250.87	5.08	271.0	309.52	14.83	258.0	269.81	9.32	246.0	254.23	4.24	240.0	242.65	1.69	241.0	242.77	2.12
b1	69	70.0	72.77	1.45	107.0	171.03	55.07	79.0	96.19	14.49	71.0	77.29	2.9	69.0	69.84	0.0	69.0	69.81	0.0
b2	76	78.0	82.42	2.63	104.0	170.42	36.84	88.0	100.16	15.79	77.0	84.45	1.32	76.0	76.77	0.0	76.0	76.81	0.0
b3	80	82.0	83.97	2.5	123.0	212.29	53.75	85.0	110.94	6.25	81.0	88.45	1.25	81.0	81.13	1.25	81.0	81.1	1.25
b4	79	83.0	86.29	5.06	120.0	193.39	51.9	89.0	104.68	12.66	83.0	88.97	5.06	79.0	81.06	0.0	79.0	81.16	0.0
b5	72	75.0	75.65	4.17	132.0	181.55	83.33	81.0	96.26	12.5	74.0	77.97	2.78	72.0	72.39	0.0	72.0	72.52	0.0
c1	227	243.0	250.42	7.05	283.0	340.26	24.67	264.0	279.74	16.3	241.0	257.63	6.17	234.0	238.42	3.08	236.0	238.65	3.96
c2	219	236.0	242.9	7.76	302.0	344.0	37.9	261.0	273.84	19.18	234.0	245.32	6.85	227.0	230.52	3.65	225.0	230.71	2.74
c3	243	259.0	264.9	6.58	332.0	398.45	36.63	274.0	297.29	12.76	259.0	272.87	6.58	247.0	251.71	1.65	249.0	252.45	2.47
c4	219	231.0	237.87	5.48	288.0	351.87	31.51	245.0	268.52	11.87	232.0	240.68	5.94	226.0	228.35	3.2	226.0	228.84	3.2
c5	215	227.0	233.81	5.58	269.0	338.32	25.12	239.0	262.68	11.16	226.0	238.39	5.12	220.0	223.1	2.33	220.0	223.65	2.33
d1	60	65.0	67.03	8.33	168.0	228.9	180.0	84.0	100.65	40.0	64.0	72.97	6.67	61.0	61.97	1.67	61.0	62.1	1.67
d2	66	68.0	71.35	3.03	183.0	258.87	177.27	84.0	115.68	27.27	69.0	76.0	4.55	67.0	67.42	1.52	66.0	67.39	0.0
d3	72	77.0	80.26	6.94	192.0	288.61	166.67	91.0	117.94	26.39	78.0	90.45	8.33	74.0	74.97	2.78	74.0	74.94	2.78
d4	62	64.0	66.35	3.23	175.0	232.74	182.26	79.0	97.45	27.42	63.0	70.87	1.61	62.0	62.39	0.0	62.0	62.74	0.0
d5	61	64.0	67.29	4.92	149.0	217.16	144.26	83.0	102.19	36.07	66.0	73.97	8.2	61.0	62.39	0.0	61.0	62.39	0.0
		263.47	269.32	4.1	295.18	341.89	34.47	270.76	286.4	9.97	261.6	271.11	3.44	257.33	261.04	1.36	257.64	261.45	1.37

**Table 12:** Results obtained with SCA and the TFBR-1, TFBR-2, TFBR-3, TFBR-4, TFBR-5 and TFBR-6 sets.

SCP		TFBR-1			TFBR-2			TFBR-3			TFBR-4			TFBR-5			TFBR-6		
**Inst.**	**Opt.**	**Best**	**Avg**	**RPD**	**Best**	**Avg**	**RPD**	**Best**	**Avg**	**RPD**	**Best**	**Avg**	**RPD**	**Best**	**Avg**	**RPD**	**Best**	**Avg**	**RPD**
41	429	436.0	442.61	1.63	626.0	666.72	45.92	446.0	461.16	3.96	630.0	669.08	46.85	431.0	434.08	0.47	430.0	434.28	0.23
42	512	545.0	554.77	6.45	1061.0	1116.68	107.23	565.0	595.16	10.35	1015.0	1095.8	98.24	524.0	530.76	2.34	525.0	531.8	2.54
43	516	537.0	549.06	4.07	1097.0	1195.08	112.6	546.0	592.72	5.81	1085.0	1210.52	110.27	522.0	526.44	1.16	520.0	527.6	0.78
44	494	519.0	530.0	5.06	881.0	975.72	78.34	537.0	562.24	8.7	857.0	964.8	73.48	499.0	508.0	1.01	503.0	508.76	1.82
45	512	537.0	550.29	4.88	1076.0	1132.76	110.16	561.0	592.44	9.57	1029.0	1113.44	100.98	521.0	526.48	1.76	522.0	527.24	1.95
46	560	577.0	590.45	3.04	1241.0	1360.88	121.61	590.0	635.92	5.36	1262.0	1372.12	125.36	564.0	568.0	0.71	564.0	568.16	0.71
47	430	438.0	449.19	1.86	796.0	853.44	85.12	457.0	482.24	6.28	779.0	853.56	81.16	434.0	436.92	0.93	432.0	437.08	0.47
48	492	505.0	514.23	2.64	1077.0	1150.96	118.9	549.0	570.76	11.59	1052.0	1160.16	113.82	494.0	499.48	0.41	495.0	500.68	0.61
49	641	682.0	695.16	6.4	1466.0	1580.72	128.71	723.0	760.64	12.79	1484.0	1590.4	131.51	660.0	672.12	2.96	661.0	672.44	3.12
410	514	526.0	534.35	2.33	978.0	1070.44	90.27	551.0	579.6	7.2	1023.0	1091.2	99.03	515.0	521.0	0.19	518.0	521.28	0.78
51	253	264.0	272.77	4.35	517.0	565.16	104.35	281.0	293.48	11.07	546.0	577.16	115.81	257.0	263.84	1.58	259.0	263.52	2.37
52	302	324.0	335.13	7.28	794.0	870.08	162.91	345.0	369.76	14.24	815.0	879.24	169.87	319.0	323.16	5.63	320.0	324.64	5.96
53	226	230.0	235.32	1.77	493.0	524.04	118.14	244.0	260.96	7.96	472.0	521.52	108.85	229.0	230.48	1.33	229.0	230.52	1.33
54	242	251.0	255.0	3.72	500.0	540.0	106.61	265.0	277.2	9.5	509.0	546.72	110.33	246.0	248.88	1.65	246.0	248.76	1.65
55	211	217.0	220.81	2.84	363.0	398.8	72.04	226.0	237.56	7.11	362.0	395.32	71.56	212.0	214.36	0.47	213.0	215.12	0.95
56	213	225.0	230.84	5.63	469.0	497.04	120.19	241.0	258.08	13.15	458.0	502.08	115.02	216.0	219.8	1.41	215.0	220.4	0.94
57	293	308.0	315.0	5.12	645.0	672.48	120.14	329.0	346.28	12.29	620.0	664.56	111.6	299.0	303.28	2.05	301.0	302.88	2.73
58	288	296.0	300.87	2.78	647.0	697.0	124.65	319.0	335.28	10.76	645.0	694.76	123.96	290.0	293.44	0.69	290.0	293.56	0.69
59	279	288.0	293.58	3.23	654.0	699.4	134.41	308.0	326.08	10.39	685.0	707.12	145.52	281.0	283.56	0.72	281.0	284.08	0.72
510	265	271.0	280.9	2.26	570.0	623.28	115.09	294.0	308.44	10.94	603.0	631.24	127.55	267.0	271.04	0.75	267.0	271.24	0.75
61	138	145.0	147.81	5.07	692.0	739.12	401.45	161.0	176.72	16.67	670.0	751.56	385.51	141.0	142.96	2.17	141.0	142.8	2.17
62	146	151.0	157.58	3.42	998.0	1098.04	583.56	181.0	216.96	23.97	1029.0	1123.04	604.79	148.0	150.8	1.37	148.0	151.2	1.37
63	145	147.0	150.77	1.38	957.0	1047.92	560.0	168.0	205.36	15.86	989.0	1048.24	582.07	146.0	148.16	0.69	146.0	148.08	0.69
64	131	134.0	135.71	2.29	602.0	649.16	359.54	147.0	160.56	12.21	595.0	656.92	354.2	131.0	132.44	0.0	131.0	132.56	0.0
65	161	168.0	181.42	4.35	1024.0	1098.32	536.02	191.0	228.28	18.63	1019.0	1099.76	532.92	164.0	167.56	1.86	162.0	167.44	0.62
a1	253	263.0	268.45	3.95	1263.0	1337.08	399.21	289.0	338.24	14.23	1233.0	1335.12	387.35	260.0	262.32	2.77	260.0	262.44	2.77
a2	252	268.0	272.65	6.35	1183.0	1228.4	369.44	282.0	335.76	11.9	1161.0	1234.96	360.71	261.0	263.4	3.57	259.0	263.6	2.78
a3	232	242.0	248.26	4.31	1066.0	1153.44	359.48	273.0	305.16	17.67	1086.0	1165.12	368.1	240.0	242.24	3.45	237.0	242.44	2.16
a4	234	246.0	251.58	5.13	1093.0	1142.04	367.09	285.0	320.56	21.79	1066.0	1148.16	355.56	238.0	241.64	1.71	240.0	243.12	2.56
a5	236	244.0	250.06	3.39	1113.0	1161.8	371.61	275.0	312.8	16.53	1086.0	1167.92	360.17	240.0	242.44	1.69	241.0	242.88	2.12
b1	69	70.0	72.26	1.45	1386.0	1463.68	1908.7	91.0	153.92	31.88	1408.0	1462.8	1940.58	69.0	69.8	0.0	69.0	70.04	0.0
b2	76	79.0	80.58	3.95	1389.0	1467.88	1727.63	106.0	155.12	39.47	1368.0	1468.24	1700.0	76.0	76.92	0.0	76.0	76.88	0.0
b3	80	82.0	82.81	2.5	1806.0	1883.88	2157.5	102.0	187.76	27.5	1818.0	1887.96	2172.5	81.0	81.08	1.25	80.0	81.04	0.0
b4	79	83.0	84.55	5.06	1560.0	1674.4	1874.68	115.0	182.4	45.57	1597.0	1676.76	1921.52	80.0	81.36	1.27	80.0	81.52	1.27
b5	72	74.0	74.65	2.78	1424.0	1495.88	1877.78	92.0	164.32	27.78	1427.0	1489.68	1881.94	72.0	72.6	0.0	72.0	72.68	0.0
c1	227	244.0	250.06	7.49	1510.0	1634.92	565.2	296.0	333.4	30.4	1555.0	1617.6	585.02	235.0	237.48	3.52	235.0	238.24	3.52
c2	219	234.0	240.48	6.85	1781.0	1863.36	713.24	286.0	329.24	30.59	246.0	1785.28	12.33	226.0	230.16	3.2	228.0	230.72	4.11
c3	243	256.0	262.06	5.35	2099.0	2182.0	763.79	319.0	370.96	31.28	1952.0	2161.6	703.29	248.0	251.32	2.06	249.0	252.32	2.47
c4	219	230.0	235.0	5.02	1635.0	1776.44	646.58	286.0	333.68	30.59	1608.0	1776.52	634.25	226.0	229.76	3.2	227.0	229.84	3.65
c5	215	226.0	232.65	5.12	1596.0	1713.16	642.33	252.0	329.96	17.21	1583.0	1707.88	636.28	221.0	223.0	2.79	219.0	222.56	1.86
d1	60	63.0	65.32	5.0	2090.0	2154.68	3383.33	90.0	174.12	50.0	2015.0	2166.6	3258.33	61.0	61.88	1.67	61.0	61.92	1.67
d2	66	68.0	69.45	3.03	2366.0	2467.0	3484.85	115.0	202.8	74.24	2369.0	2460.32	3489.39	67.0	67.4	1.52	67.0	67.2	1.52
d3	72	76.0	78.16	5.56	2587.0	2705.04	3493.06	99.0	213.32	37.5	2611.0	2691.96	3526.39	74.0	75.0	2.78	74.0	74.72	2.78
d4	62	63.0	63.61	1.61	2089.0	2192.56	3269.35	91.0	171.88	46.77	2075.0	2164.4	3246.77	62.0	62.76	0.0	62.0	62.84	0.0
d5	61	64.0	65.71	4.92	2119.0	2199.12	3373.77	82.0	167.12	34.43	2072.0	2181.68	3296.72	61.0	62.24	0.0	61.0	62.12	0.0
		264.36	270.49	4.06	1186.2	1260.44	808.15	290.02	331.48	20.3	1145.98	1259.35	788.39	257.96	261.15	1.57	258.13	261.45	1.58

**Table 13:** Continued results obtained with SCA and the TFBR-7, TFBR-8, TFBR-9, TFBR-10, TFBR-11 and TFBR-12 sets.

SCP		TFBR-7			TFBR-8			TFBR-9			TFBR-10			TFBR-11			TFBR-12		
**Inst.**	**Opt.**	**Best**	**Avg**	**RPD**	**Best**	**Avg**	**RPD**	**Best**	**Avg**	**RPD**	**Best**	**Avg**	**RPD**	**Best**	**Avg**	**RPD**	**Best**	**Avg**	**RPD**
41	429	438.0	443.0	2.1	632.0	672.92	47.32	434.0	442.32	1.17	620.0	671.56	44.52	432.0	435.0	0.7	430.0	434.64	0.23
42	512	541.0	551.16	5.66	1023.0	1105.32	99.8	537.0	552.44	4.88	1051.0	1120.24	105.27	522.0	531.68	1.95	526.0	533.88	2.73
43	516	538.0	546.71	4.26	1134.0	1194.16	119.77	533.0	546.96	3.29	1148.0	1210.96	122.48	524.0	529.56	1.55	523.0	530.08	1.36
44	494	515.0	528.97	4.25	904.0	967.88	83.0	516.0	527.28	4.45	875.0	965.72	77.13	501.0	508.6	1.42	503.0	510.08	1.82
45	512	531.0	549.19	3.71	1072.0	1138.52	109.38	526.0	543.4	2.73	1042.0	1117.36	103.52	522.0	528.2	1.95	522.0	528.64	1.95
46	560	573.0	586.55	2.32	1288.0	1377.0	130.0	575.0	584.76	2.68	1269.0	1365.32	126.61	564.0	569.08	0.71	566.0	568.84	1.07
47	430	440.0	447.65	2.33	790.0	865.24	83.72	441.0	449.12	2.56	790.0	864.6	83.72	436.0	438.12	1.4	434.0	438.16	0.93
48	492	505.0	513.0	2.64	1086.0	1163.44	120.73	503.0	514.08	2.24	1077.0	1160.92	118.9	496.0	500.28	0.81	497.0	500.84	1.02
49	641	679.0	692.35	5.93	1504.0	1592.68	134.63	686.0	700.72	7.02	1473.0	1560.2	129.8	663.0	673.72	3.43	660.0	674.64	2.96
410	514	527.0	533.74	2.53	1004.0	1079.0	95.33	526.0	534.64	2.33	972.0	1053.84	89.11	519.0	522.04	0.97	518.0	522.4	0.78
51	253	264.0	271.1	4.35	502.0	575.84	98.42	269.0	274.4	6.32	548.0	575.16	116.6	261.0	263.92	3.16	259.0	264.24	2.37
52	302	327.0	333.97	8.28	811.0	870.52	168.54	330.0	335.8	9.27	815.0	872.24	169.87	318.0	325.16	5.3	318.0	324.2	5.3
53	226	233.0	234.9	3.1	491.0	527.4	117.26	231.0	236.36	2.21	488.0	521.72	115.93	230.0	230.56	1.77	230.0	230.88	1.77
54	242	249.0	254.06	2.89	510.0	544.64	110.74	251.0	254.76	3.72	506.0	541.84	109.09	247.0	249.48	2.07	244.0	248.96	0.83
55	211	217.0	219.45	2.84	358.0	397.68	69.67	217.0	221.36	2.84	356.0	398.04	68.72	213.0	215.76	0.95	213.0	215.16	0.95
56	213	221.0	229.61	3.76	483.0	502.12	126.76	228.0	231.08	7.04	427.0	497.88	100.47	217.0	221.2	1.88	216.0	220.88	1.41
57	293	307.0	313.97	4.78	616.0	663.68	110.24	307.0	314.0	4.78	642.0	673.6	119.11	298.0	303.84	1.71	295.0	303.32	0.68
58	288	295.0	300.16	2.43	633.0	691.92	119.79	298.0	303.0	3.47	673.0	700.72	133.68	291.0	294.12	1.04	292.0	294.12	1.39
59	279	286.0	291.81	2.51	652.0	695.36	133.69	288.0	293.52	3.23	627.0	693.0	124.73	282.0	284.64	1.08	281.0	284.76	0.72
510	265	275.0	279.87	3.77	552.0	617.12	108.3	271.0	279.0	2.26	609.0	632.04	129.81	267.0	271.88	0.75	268.0	271.36	1.13
61	138	143.0	147.1	3.62	667.0	741.8	383.33	144.0	148.76	4.35	689.0	740.64	399.28	140.0	143.16	1.45	141.0	143.04	2.17
62	146	152.0	157.03	4.11	1039.0	1111.2	611.64	154.0	157.6	5.48	935.0	1099.08	540.41	149.0	151.32	2.05	148.0	150.64	1.37
63	145	147.0	150.48	1.38	977.0	1046.24	573.79	149.0	152.4	2.76	958.0	1042.36	560.69	147.0	148.24	1.38	147.0	148.52	1.38
64	131	132.0	135.32	0.76	606.0	651.44	362.6	134.0	136.12	2.29	608.0	655.08	364.12	131.0	132.72	0.0	132.0	132.96	0.76
65	161	175.0	181.48	8.7	982.0	1095.04	509.94	172.0	178.4	6.83	1008.0	1084.64	526.09	162.0	168.92	0.62	162.0	168.68	0.62
a1	253	263.0	267.77	3.95	1279.0	1349.96	405.53	266.0	270.16	5.14	1236.0	1327.92	388.54	259.0	262.56	2.37	261.0	262.84	3.16
a2	252	264.0	271.68	4.76	1134.0	1221.84	350.0	268.0	274.0	6.35	1161.0	1232.48	360.71	262.0	264.08	3.97	260.0	264.12	3.17
a3	232	242.0	247.84	4.31	1100.0	1168.4	374.14	247.0	250.32	6.47	1071.0	1152.0	361.64	239.0	242.52	3.02	241.0	242.52	3.88
a4	234	242.0	250.52	3.42	1066.0	1133.2	355.56	250.0	253.48	6.84	1078.0	1141.72	360.68	241.0	243.12	2.99	240.0	243.24	2.56
a5	236	246.0	250.0	4.24	1084.0	1172.12	359.32	248.0	250.92	5.08	1132.0	1168.44	379.66	241.0	242.84	2.12	240.0	242.84	1.69
b1	69	70.0	72.29	1.45	1345.0	1440.92	1849.28	71.0	72.56	2.9	1282.0	1445.64	1757.97	69.0	70.12	0.0	69.0	70.04	0.0
b2	76	79.0	80.45	3.95	1364.0	1471.2	1694.74	79.0	82.56	3.95	1352.0	1449.48	1678.95	76.0	76.84	0.0	76.0	76.96	0.0
b3	80	82.0	82.68	2.5	1823.0	1870.48	2178.75	83.0	84.68	3.75	1732.0	1857.12	2065.0	81.0	81.08	1.25	80.0	81.24	0.0
b4	79	83.0	84.68	5.06	1604.0	1678.4	1930.38	85.0	86.32	7.59	1614.0	1681.24	1943.04	80.0	81.72	1.27	80.0	81.64	1.27
b5	72	74.0	74.74	2.78	1396.0	1485.4	1838.89	74.0	75.68	2.78	1334.0	1486.04	1752.78	72.0	72.56	0.0	72.0	72.44	0.0
c1	227	239.0	248.26	5.29	1556.0	1624.12	585.46	244.0	250.68	7.49	1511.0	1627.56	565.64	233.0	238.2	2.64	235.0	238.6	3.52
c2	219	229.0	239.71	4.57	1733.0	1848.56	691.32	237.0	241.8	8.22	1778.0	1859.8	711.87	226.0	230.76	3.2	228.0	230.64	4.11
c3	243	253.0	261.42	4.12	2092.0	2189.8	760.91	256.0	265.56	5.35	2090.0	2171.96	760.08	248.0	252.4	2.06	247.0	252.48	1.65
c4	219	232.0	234.84	5.94	1678.0	1787.12	666.21	234.0	239.28	6.85	1709.0	1780.92	680.37	228.0	230.32	4.11	225.0	230.2	2.74
c5	215	224.0	231.58	4.19	1649.0	1712.48	666.98	226.0	232.32	5.12	1633.0	1723.8	659.53	221.0	223.36	2.79	221.0	223.4	2.79
d1	60	64.0	65.58	6.67	2067.0	2163.96	3345.0	64.0	66.88	6.67	2017.0	2164.88	3261.67	61.0	62.04	1.67	61.0	62.2	1.67
d2	66	68.0	69.39	3.03	2383.0	2466.44	3510.61	70.0	72.4	6.06	2314.0	2455.12	3406.06	66.0	67.48	0.0	67.0	67.36	1.52
d3	72	77.0	78.03	6.94	2623.0	2699.56	3543.06	77.0	80.32	6.94	2372.0	2687.96	3194.44	73.0	74.92	1.39	73.0	74.92	1.39
d4	62	62.0	63.71	0.0	2078.0	2197.04	3251.61	63.0	66.16	1.61	2057.0	2208.84	3217.74	62.0	62.84	0.0	62.0	63.0	0.0
d5	61	64.0	65.74	4.92	2055.0	2173.96	3268.85	65.0	67.76	6.56	2080.0	2187.8	3309.84	62.0	62.44	1.64	61.0	62.52	0.0
		263.71	269.63	3.89	1187.22	1260.96	805.67	265.04	271.02	4.71	1172.42	1258.43	786.57	258.49	261.85	1.7	258.31	261.94	1.62

**Table 14:** Results obtained with WOA and the TFBR-1, TFBR-2, TFBR-3, TFBR-4, TFBR-5, and TFBR-6 sets.

SCP		TFBR-1			TFBR-2			TFBR-3			TFBR-4			TFBR-5			TFBR-6		
**Inst.**	**Opt.**	**Best**	**Avg**	**RPD**	**Best**	**Avg**	**RPD**	**Best**	**Avg**	**RPD**	**Best**	**Avg**	**RPD**	**Best**	**Avg**	**RPD**	**Best**	**Avg**	**RPD**
41	429	432.0	439.13	0.7	434.0	472.16	1.17	449.0	466.03	4.66	437.0	472.77	1.86	431.0	434.77	0.47	430.0	434.19	0.23
42	512	540.0	546.39	5.47	553.0	634.94	8.01	575.0	602.9	12.3	539.0	637.65	5.27	521.0	531.0	1.76	520.0	531.52	1.56
43	516	531.0	542.9	2.91	551.0	645.39	6.78	578.0	612.65	12.02	541.0	636.1	4.84	521.0	525.65	0.97	520.0	527.03	0.78
44	494	510.0	523.16	3.24	526.0	591.13	6.48	544.0	573.06	10.12	519.0	568.45	5.06	500.0	507.77	1.21	501.0	509.16	1.42
45	512	533.0	543.16	4.1	554.0	641.94	8.2	570.0	602.97	11.33	539.0	665.68	5.27	520.0	526.87	1.56	521.0	525.74	1.76
46	560	569.0	582.45	1.61	607.0	697.77	8.39	597.0	649.13	6.61	591.0	725.03	5.54	562.0	568.84	0.36	565.0	569.48	0.89
47	430	438.0	445.42	1.86	442.0	506.23	2.79	457.0	489.16	6.28	448.0	506.9	4.19	435.0	437.42	1.16	435.0	437.55	1.16
48	492	499.0	507.48	1.42	528.0	622.26	7.32	547.0	587.9	11.18	527.0	607.55	7.11	494.0	500.48	0.41	493.0	500.23	0.2
49	641	670.0	688.06	4.52	736.0	860.45	14.82	731.0	775.23	14.04	707.0	841.1	10.3	662.0	672.9	3.28	663.0	673.48	3.43
410	514	523.0	530.52	1.75	529.0	623.35	2.92	561.0	590.55	9.14	545.0	619.13	6.03	518.0	522.1	0.78	515.0	521.84	0.19
51	253	261.0	267.94	3.16	279.0	327.26	10.28	288.0	301.81	13.83	277.0	312.84	9.49	258.0	264.03	1.98	258.0	263.19	1.98
52	302	324.0	332.03	7.28	345.0	417.77	14.24	350.0	377.74	15.89	344.0	401.81	13.91	315.0	323.29	4.3	316.0	323.55	4.64
53	226	231.0	233.84	2.21	239.0	288.39	5.75	243.0	266.26	7.52	247.0	283.77	9.29	229.0	230.48	1.33	229.0	230.74	1.33
54	242	249.0	252.81	2.89	266.0	305.58	9.92	265.0	282.55	9.5	258.0	302.26	6.61	246.0	248.74	1.65	246.0	248.26	1.65
55	211	217.0	218.71	2.84	216.0	246.06	2.37	227.0	242.16	7.58	227.0	251.29	7.58	212.0	215.35	0.47	212.0	215.13	0.47
56	213	222.0	227.61	4.23	235.0	277.29	10.33	235.0	259.45	10.33	230.0	273.74	7.98	214.0	219.45	0.47	217.0	220.32	1.88
57	293	305.0	311.65	4.1	316.0	370.39	7.85	325.0	349.74	10.92	318.0	364.39	8.53	297.0	302.45	1.37	297.0	303.48	1.37
58	288	295.0	297.97	2.43	316.0	373.1	9.72	325.0	342.74	12.85	308.0	374.19	6.94	291.0	294.13	1.04	289.0	293.77	0.35
59	279	283.0	289.13	1.43	306.0	357.52	9.68	314.0	333.42	12.54	312.0	358.39	11.83	280.0	284.74	0.36	281.0	284.32	0.72
510	265	274.0	278.81	3.4	285.0	344.55	7.55	300.0	316.94	13.21	292.0	334.42	10.19	267.0	271.19	0.75	267.0	271.13	0.75
61	138	142.0	146.32	2.9	154.0	194.13	11.59	154.0	184.81	11.59	153.0	200.48	10.87	140.0	142.61	1.45	142.0	142.94	2.9
62	146	152.0	156.32	4.11	174.0	252.29	19.18	172.0	224.0	17.81	159.0	208.23	8.9	147.0	150.55	0.68	148.0	150.52	1.37
63	145	148.0	150.29	2.07	155.0	225.52	6.9	173.0	220.03	19.31	173.0	253.19	19.31	146.0	148.52	0.69	147.0	148.55	1.38
64	131	133.0	135.1	1.53	137.0	165.84	4.58	145.0	176.1	10.69	142.0	177.68	8.4	131.0	132.73	0.0	131.0	132.97	0.0
65	161	170.0	178.74	5.59	185.0	255.1	14.91	206.0	243.74	27.95	196.0	267.97	21.74	163.0	167.68	1.24	161.0	168.32	0.0
a1	253	263.0	267.19	3.95	331.0	417.9	30.83	312.0	353.61	23.32	323.0	466.9	27.67	259.0	262.32	2.37	260.0	262.35	2.77
a2	252	266.0	270.16	5.56	315.0	458.42	25.0	300.0	348.48	19.05	311.0	422.13	23.41	259.0	264.03	2.78	260.0	264.29	3.17
a3	232	242.0	246.68	4.31	278.0	368.35	19.83	276.0	318.16	18.97	292.0	346.03	25.86	239.0	242.61	3.02	239.0	242.74	3.02
a4	234	244.0	249.58	4.27	299.0	412.32	27.78	282.0	327.13	20.51	308.0	431.13	31.62	239.0	242.9	2.14	239.0	242.81	2.14
a5	236	244.0	248.61	3.39	289.0	361.35	22.46	266.0	330.26	12.71	308.0	405.71	30.51	241.0	242.84	2.12	241.0	243.29	2.12
b1	69	70.0	71.65	1.45	111.0	312.71	60.87	93.0	175.65	34.78	126.0	259.13	82.61	69.0	70.06	0.0	69.0	69.94	0.0
b2	76	77.0	80.1	1.32	140.0	255.65	84.21	134.0	185.74	76.32	129.0	295.87	69.74	76.0	77.1	0.0	76.0	76.68	0.0
b3	80	81.0	82.61	1.25	161.0	318.26	101.25	105.0	207.81	31.25	165.0	305.42	106.25	81.0	81.39	1.25	80.0	81.23	0.0
b4	79	82.0	84.16	3.8	132.0	323.35	67.09	129.0	206.81	63.29	146.0	332.26	84.81	79.0	81.19	0.0	79.0	81.16	0.0
b5	72	74.0	74.52	2.78	124.0	230.61	72.22	118.0	184.29	63.89	140.0	320.29	94.44	72.0	72.48	0.0	72.0	72.71	0.0
c1	227	243.0	247.77	7.05	356.0	529.97	56.83	289.0	364.35	27.31	376.0	529.68	65.64	235.0	238.48	3.52	234.0	238.03	3.08
c2	219	236.0	239.19	7.76	365.0	508.9	66.67	305.0	371.13	39.27	326.0	462.74	48.86	224.0	230.26	2.28	227.0	230.1	3.65
c3	243	255.0	259.65	4.94	415.0	553.03	70.78	338.0	399.61	39.09	403.0	649.06	65.84	249.0	252.84	2.47	248.0	252.58	2.06
c4	219	229.0	234.13	4.57	349.0	491.23	59.36	275.0	361.42	25.57	353.0	559.61	61.19	227.0	229.97	3.65	225.0	229.81	2.74
c5	215	227.0	230.65	5.58	310.0	473.52	44.19	275.0	341.94	27.91	330.0	545.84	53.49	221.0	223.45	2.79	220.0	223.74	2.33
d1	60	62.0	64.74	3.33	210.0	527.0	250.0	111.0	222.45	85.0	192.0	472.55	220.0	61.0	61.84	1.67	60.0	61.9	0.0
d2	66	68.0	69.0	3.03	291.0	697.77	340.91	147.0	251.94	122.73	289.0	755.23	337.88	66.0	67.52	0.0	67.0	67.55	1.52
d3	72	76.0	77.42	5.56	319.0	781.61	343.06	104.0	277.29	44.44	300.0	688.87	316.67	73.0	74.94	1.39	73.0	75.0	1.39
d4	62	62.0	63.52	0.0	234.0	610.23	277.42	101.0	215.94	62.9	226.0	569.26	264.52	62.0	62.9	0.0	62.0	62.97	0.0
d5	61	63.0	65.48	3.28	227.0	575.29	272.13	134.0	241.35	119.67	198.0	422.32	224.59	61.0	62.45	0.0	61.0	62.48	0.0
		262.56	267.84	3.44	318.31	442.31	55.66	298.33	350.14	27.94	317.11	441.89	54.5	257.62	261.45	1.36	257.69	261.53	1.39

**Table 15:** Continued results obtained with WOA and the TFBR-7, TFBR-8, TFBR-9, TFBR-10, TFBR-11, and TFBR-12 sets.

SCP		TFBR-7			TFBR-8			TFBR-9			TFBR-10			TFBR-11			TFBR-12		
**Inst.**	**Opt.**	**Best**	**Avg**	**RPD**	**Best**	**Avg**	**RPD**	**Best**	**Avg**	**RPD**	**Best**	**Avg**	**RPD**	**Best**	**Avg**	**RPD**	**Best**	**Avg**	**RPD**
41	429	434.0	439.42	1.17	611.0	647.0	42.42	434.0	439.65	1.17	556.0	637.68	29.6	430.0	435.42	0.23	432.0	435.16	0.7
42	512	538.0	546.74	5.08	649.0	1037.35	26.76	528.0	545.06	3.12	912.0	1029.32	78.12	523.0	534.13	2.15	529.0	535.39	3.32
43	516	527.0	542.87	2.13	1076.0	1143.58	108.53	532.0	542.23	3.1	997.0	1137.03	93.22	525.0	530.23	1.74	523.0	529.74	1.36
44	494	509.0	522.29	3.04	865.0	934.58	75.1	508.0	520.68	2.83	847.0	922.74	71.46	500.0	508.74	1.21	495.0	511.1	0.2
45	512	530.0	543.77	3.52	897.0	1067.16	75.2	529.0	538.55	3.32	950.0	1080.19	85.55	521.0	529.39	1.76	523.0	528.03	2.15
46	560	565.0	581.13	0.89	1174.0	1305.71	109.64	569.0	576.0	1.61	1171.0	1296.94	109.11	566.0	570.13	1.07	566.0	569.68	1.07
47	430	438.0	444.84	1.86	739.0	813.0	71.86	437.0	444.74	1.63	748.0	803.19	73.95	434.0	437.55	0.93	434.0	438.1	0.93
48	492	501.0	508.68	1.83	989.0	1088.74	101.02	498.0	508.55	1.22	1002.0	1101.55	103.66	497.0	501.29	1.02	493.0	500.32	0.2
49	641	676.0	688.23	5.46	1263.0	1492.29	97.04	673.0	691.94	4.99	1333.0	1496.29	107.96	665.0	676.57	3.74	665.0	679.16	3.74
410	514	524.0	531.1	1.95	914.0	1018.39	77.82	517.0	528.68	0.58	905.0	1024.03	76.07	517.0	522.26	0.58	520.0	523.13	1.17
51	253	261.0	268.19	3.16	495.0	547.19	95.65	264.0	270.9	4.35	506.0	546.23	100.0	259.0	264.74	2.37	261.0	265.16	3.16
52	302	323.0	330.61	6.95	743.0	836.42	146.03	324.0	331.32	7.28	748.0	828.71	147.68	318.0	324.9	5.3	317.0	324.29	4.97
53	226	231.0	233.97	2.21	446.0	501.48	97.35	230.0	234.06	1.77	465.0	496.48	105.75	229.0	230.68	1.33	229.0	230.94	1.33
54	242	249.0	252.13	2.89	484.0	514.68	100.0	248.0	253.06	2.48	468.0	513.77	93.39	246.0	249.03	1.65	247.0	249.39	2.07
55	211	214.0	217.65	1.42	317.0	371.71	50.24	214.0	218.71	1.42	331.0	377.32	56.87	212.0	215.52	0.47	214.0	215.42	1.42
56	213	220.0	226.29	3.29	448.0	474.71	110.33	222.0	227.0	4.23	416.0	473.1	95.31	216.0	221.03	1.41	218.0	221.39	2.35
57	293	305.0	311.42	4.1	603.0	642.48	105.8	305.0	310.65	4.1	573.0	632.74	95.56	300.0	304.32	2.39	297.0	303.74	1.37
58	288	296.0	298.71	2.78	543.0	663.23	88.54	291.0	298.77	1.04	603.0	658.77	109.38	291.0	294.65	1.04	291.0	293.71	1.04
59	279	285.0	289.26	2.15	598.0	658.23	114.34	286.0	289.87	2.51	559.0	658.32	100.36	281.0	284.48	0.72	282.0	284.35	1.08
510	265	273.0	278.03	3.02	517.0	595.39	95.09	271.0	277.35	2.26	531.0	593.97	100.38	268.0	272.29	1.13	267.0	271.42	0.75
61	138	145.0	146.19	5.07	657.0	705.68	376.09	143.0	146.52	3.62	588.0	694.32	326.09	142.0	143.58	2.9	141.0	143.26	2.17
62	146	150.0	155.87	2.74	853.0	1051.29	484.25	152.0	155.45	4.11	890.0	1044.55	509.59	148.0	151.77	1.37	149.0	151.39	2.05
63	145	147.0	150.45	1.38	735.0	981.26	406.9	147.0	150.39	1.38	919.0	1005.35	533.79	146.0	148.68	0.69	146.0	148.42	0.69
64	131	133.0	134.9	1.53	539.0	616.9	311.45	131.0	134.65	0.0	501.0	603.61	282.44	131.0	132.77	0.0	131.0	133.16	0.0
65	161	171.0	178.9	6.21	756.0	1041.06	369.57	171.0	176.68	6.21	878.0	1046.81	445.34	164.0	169.58	1.86	166.0	170.35	3.11
a1	253	263.0	266.42	3.95	1163.0	1289.55	359.68	262.0	266.84	3.56	1158.0	1279.52	357.71	261.0	262.77	3.16	261.0	262.97	3.16
a2	252	264.0	271.06	4.76	1057.0	1186.23	319.44	265.0	270.39	5.16	1076.0	1187.03	326.98	261.0	264.39	3.57	261.0	264.39	3.57
a3	232	243.0	246.97	4.74	978.0	1101.74	321.55	244.0	246.65	5.17	1030.0	1110.61	343.97	241.0	243.26	3.88	240.0	242.9	3.45
a4	234	241.0	248.48	2.99	965.0	1095.94	312.39	243.0	249.35	3.85	964.0	1086.9	311.97	238.0	243.23	1.71	238.0	243.58	1.71
a5	236	242.0	247.65	2.54	1045.0	1124.58	342.8	243.0	247.84	2.97	1010.0	1123.42	327.97	240.0	243.1	1.69	240.0	243.42	1.69
b1	69	71.0	72.0	2.9	1316.0	1414.39	1807.25	70.0	71.81	1.45	1318.0	1409.9	1810.14	69.0	69.9	0.0	69.0	69.9	0.0
b2	76	78.0	79.71	2.63	1278.0	1414.03	1581.58	77.0	79.68	1.32	1302.0	1421.52	1613.16	76.0	77.1	0.0	76.0	76.9	0.0
b3	80	81.0	82.35	1.25	1701.0	1826.77	2026.25	81.0	83.23	1.25	1687.0	1823.32	2008.75	80.0	81.03	0.0	80.0	81.26	0.0
b4	79	81.0	83.81	2.53	1506.0	1635.9	1806.33	82.0	83.71	3.8	1420.0	1611.94	1697.47	80.0	81.9	1.27	80.0	81.87	1.27
b5	72	73.0	74.35	1.39	1300.0	1452.1	1705.56	73.0	74.48	1.39	1216.0	1428.77	1588.89	72.0	72.65	0.0	72.0	72.71	0.0
c1	227	239.0	246.1	5.29	1483.0	1585.42	553.3	239.0	244.77	5.29	1434.0	1592.42	531.72	235.0	238.45	3.52	235.0	238.23	3.52
c2	219	229.0	238.9	4.57	1227.0	1761.81	460.27	234.0	237.65	6.85	1629.0	1779.84	643.84	228.0	231.19	4.11	228.0	231.32	4.11
c3	243	255.0	259.68	4.94	1825.0	2100.68	651.03	248.0	259.52	2.06	1916.0	2113.77	688.48	249.0	252.84	2.47	249.0	252.94	2.47
c4	219	230.0	233.9	5.02	1608.0	1730.81	634.25	227.0	235.0	3.65	1616.0	1731.35	637.9	226.0	229.9	3.2	227.0	230.06	3.65
c5	215	224.0	229.61	4.19	1590.0	1663.94	639.53	226.0	228.94	5.12	1599.0	1681.77	643.72	221.0	223.52	2.79	220.0	223.16	2.33
d1	60	62.0	64.9	3.33	1929.0	2091.71	3115.0	62.0	64.68	3.33	2019.0	2122.1	3265.0	61.0	62.26	1.67	61.0	62.39	1.67
d2	66	68.0	69.13	3.03	2010.0	2395.26	2945.45	68.0	69.42	3.03	2255.0	2433.26	3316.67	67.0	67.65	1.52	67.0	67.68	1.52
d3	72	76.0	77.77	5.56	2323.0	2629.03	3126.39	76.0	77.71	5.56	2408.0	2622.19	3244.44	74.0	75.32	2.78	74.0	75.55	2.78
d4	62	63.0	63.68	1.61	868.0	2064.03	1300.0	63.0	64.1	1.61	1962.0	2136.19	3064.52	62.0	62.84	0.0	62.0	62.77	0.0
d5	61	63.0	64.97	3.28	1962.0	2125.19	3116.39	62.0	64.94	1.64	2013.0	2107.77	3200.0	62.0	62.58	1.64	61.0	62.68	0.0
		262.02	267.62	3.25	1045.44	1209.75	685.81	261.53	267.38	3.08	1098.42	1211.26	745.64	258.49	262.21	1.73	258.6	262.29	1.76

**Table 16:** Ranking of the best sets considering RPD for GWO.

1. TFBR-5	4. TFBR-6	7. TFBR-7	10. TFBR-3
2. TFBR-11	5. TFBR-10	8. TFBR-4	11. TFBR-2
3. TFBR-12	6. TFBR-1	9. TFBR-9	12. TFBR-8

**Table 17:** Ranking of the best sets considering RPD for SCA.

1. TFBR-6	4. TFBR-11	7. TFBR-9	10. TFBR-4
2. TFBR-5	5. TFBR-7	8. TFBR-3	11. TFBR-8
3. TFBR-12	6. TFBR-1	9. TFBR-2	12. TFBR-10

**Table 18:** Ranking of the best sets considering RPD for WOA.

1. TFBR-6	4. TFBR-11	7. TFBR-7	10. TFBR-3
2. TFBR-5	5. TFBR-9	8. TFBR-4	11. TFBR-8
3. TFBR-12	6. TFBR-1	9. TFBR-2	12. TFBR-10

**Table 19:** Ranking of the best sets considering fitness obtained using GWO for scp44 instance.

Set—Fitness	Set—Fitness	Set—Fitness	Set—Fitness
1. TFBR-5—496	4. TFBR-6—502	7. TFBR-1—508	10. TFBR-2—520
2. TFBR-11—499	5. TFBR-4—506	8. TFBR-9—513	11. TFBR-3—523
3. TFBR-12—501	6. TFBR-10—506	9. TFBR-7—517	12. TFBR-8—528

**Table 20:** Ranking of the best sets considering fitness obtained using SCA for scpb2 instance.

Set—Fitness	Set—Fitness	Set—Fitness	Set—Fitness
1. TFBR-5—76	4. TFBR-12—76	7. TFBR-9—79	10. TFBR-8—1364
2. TFBR-6—76	5. TFBR-1—79	8. TFBR-3—106	11. TFBR-4—1368
3. TFBR-11—76	6. TFBR-7—79	9. TFBR-10—1352	12. TFBR-2—1389

**Table 21:** Ranking of the best sets considering fitness obtained using WOA for scp65 instance.

Set—Fitness	Set—Fitness	Set—Fitness	Set—Fitness
1. TFBR-6—161	4. TFBR-12—166	7. TFBR-9—171	10. TFBR-3—206
2. TFBR-5—163	5. TFBR-1—170	8. TFBR-2—185	11. TFBR-8—756
3. TFBR-11—164	6. TFBR-7—171	9. TFBR-4—196	12. TFBR-10—878

**Table 22:** Average *p*-value of GWO compared to others algorithm.

	TFBR-1	TFBR-2	TFBR-3	TFBR-4	TFBR-5	TFBR-6	TFBR-7	TFBR-8	TFBR-9	TFBR-10	TFBR-11	TFBR-12
TFBR-1	-	**0.00**	**0.00**	**0.00**	≥0.05	≥0.05	≥0.05	**0.00**	**0.00**	≥0.05	≥0.05	≥0.05
TFBR-2	≥0.05	-	≥0.05	≥0.05	≥0.05	≥0.05	≥0.05	≥0.05	≥0.05	≥0.05	≥0.05	≥0.05
TFBR-3	≥0.05	**0.00**	-	≥0.05	≥0.05	≥0.05	≥0.05	**0.00**	≥0.05	≥0.05	≥0.05	≥0.05
TFBR-4	≥0.05	**0.00**	**0.00**	-	≥0.05	≥0.05	≥0.05	**0.00**	≥0.05	≥0.05	≥0.05	≥0.05
TFBR-5	**0.00**	**0.00**	**0.00**	**0.00**	-	≥0.05	**0.00**	**0.00**	**0.00**	**0.00**	≥0.05	≥0.05
TFBR-6	**0.00**	**0.00**	**0.00**	**0.00**	≥0.05	-	**0.00**	**0.00**	**0.00**	**0.00**	≥0.05	≥0.05
TFBR-7	≥0.05	**0.00**	**0.00**	**0.00**	≥0.05	≥0.05	-	**0.00**	**0.00**	≥0.05	≥0.05	≥0.05
TFBR-8	≥0.05	**0.02**	≥0.05	≥0.05	≥0.05	≥0.05	≥0.05	-	≥0.05	≥0.05	≥0.05	≥0.05
TFBR-9	≥0.05	**0.00**	**0.00**	≥0.05	≥0.05	≥0.05	≥0.05	**0.00**	-	≥0.05	≥0.05	≥0.05
TFBR-10	≥0.05	**0.00**	**0.00**	**0.03**	≥0.05	≥0.05	≥0.05	**0.00**	**0.00**	-	≥0.05	≥0.05
TFBR-11	**0.00**	**0.00**	**0.00**	**0.00**	≥0.05	≥0.05	**0.00**	**0.00**	**0.00**	**0.00**	-	≥0.05
TFBR-12	**0.00**	**0.00**	**0.00**	**0.00**	≥0.05	≥0.05	**0.00**	**0.00**	**0.00**	**0.00**	≥0.05	-

**Table 23:** Average *p*-value of SCA compared to others algorithm.

	TFBR-1	TFBR-2	TFBR-3	TFBR-4	TFBR-5	TFBR-6	TFBR-7	TFBR-8	TFBR-9	TFBR-10	TFBR-11	TFBR-12
TFBR-1	-	**0.00**	**0.00**	**0.00**	≥0.05	≥0.05	≥0.05	**0.00**	≥0.05	**0.00**	≥0.05	≥0.05
TFBR-2	≥0.05	-	≥0.05	≥0.05	≥0.05	≥0.05	≥0.05	≥0.05	≥0.05	≥0.05	≥0.05	≥0.05
TFBR-3	≥0.05	**0.00**	-	**0.00**	≥0.05	≥0.05	≥0.05	**0.00**	≥0.05	**0.00**	≥0.05	≥0.05
TFBR-4	≥0.05	≥0.05	≥0.05	-	≥0.05	≥0.05	≥0.05	≥0.05	≥0.05	≥0.05	≥0.05	≥0.05
TFBR-5	**0.00**	**0.00**	**0.00**	**0.00**	-	≥0.05	**0.00**	**0.00**	**0.00**	**0.00**	≥0.05	≥0.05
TFBR-6	**0.00**	**0.00**	**0.00**	**0.00**	≥0.05	-	**0.00**	**0.00**	**0.00**	**0.00**	≥0.05	≥0.05
TFBR-7	≥0.05	**0.00**	**0.00**	**0.00**	≥0.05	≥0.05	-	**0.00**	≥0.05	**0.00**	≥0.05	≥0.05
TFBR-8	≥0.05	≥0.05	≥0.05	≥0.05	≥0.05	≥0.05	≥0.05	-	≥0.05	≥0.05	≥0.05	≥0.05
TFBR-9	≥0.05	**0.00**	**0.00**	**0.00**	≥0.05	≥0.05	≥0.05	**0.00**	-	**0.00**	≥0.05	≥0.05
TFBR-10	≥0.05	≥0.05	≥0.05	≥0.05	≥0.05	≥0.05	≥0.05	≥0.05	≥0.05	-	≥0.05	≥0.05
TFBR-11	**0.00**	**0.00**	**0.00**	**0.00**	≥0.05	≥0.05	**0.00**	**0.00**	**0.00**	**0.00**	-	≥0.05
TFBR-12	**0.00**	**0.00**	**0.00**	**0.00**	≥0.05	≥0.05	**0.00**	**0.00**	**0.00**	**0.00**	≥0.05	-

**Table 24:** Average *p*-value of WOA compared to others algorithm.

	TFBR-1	TFBR-2	TFBR-3	TFBR-4	TFBR-5	TFBR-6	TFBR-7	TFBR-8	TFBR-9	TFBR-10	TFBR-11	TFBR-12
TFBR-1	-	**0.00**	**0.00**	**0.00**	≥0.05	≥0.05	≥0.05	**0.00**	≥0.05	**0.00**	≥0.05	≥0.05
TFBR-2	≥0.05	-	≥0.05	≥0.05	≥0.05	≥0.05	≥0.05	**0.00**	≥0.05	**0.00**	≥0.05	≥0.05
TFBR-3	≥0.05	≥0.05	-	≥0.05	≥0.05	≥0.05	≥0.05	**0.00**	≥0.05	**0.00**	≥0.05	≥0.05
TFBR-4	≥0.05	≥0.05	≥0.05	-	≥0.05	≥0.05	≥0.05	**0.00**	≥0.05	**0.00**	≥0.05	≥0.05
TFBR-5	**0.00**	**0.00**	**0.00**	**0.00**	-	≥0.05	**0.00**	**0.00**	**0.00**	**0.00**	≥0.05	≥0.05
TFBR-6	**0.00**	**0.00**	**0.00**	**0.00**	≥0.05	-	**0.00**	**0.00**	**0.00**	**0.00**	≥0.05	≥0.05
TFBR-7	≥0.05	**0.00**	**0.00**	**0.00**	≥0.05	≥0.05	-	**0.00**	≥0.05	**0.00**	≥0.05	≥0.05
TFBR-8	≥0.05	≥0.05	≥0.05	≥0.05	≥0.05	≥0.05	≥0.05	-	≥0.05	≥0.05	≥0.05	≥0.05
TFBR-9	≥0.05	**0.00**	**0.00**	**0.00**	≥0.05	≥0.05	≥0.05	**0.00**	-	**0.00**	≥0.05	≥0.05
TFBR-10	≥0.05	≥0.05	≥0.05	≥0.05	≥0.05	≥0.05	≥0.05	≥0.05	≥0.05	-	≥0.05	≥0.05
TFBR-11	**0.00**	**0.00**	**0.00**	**0.00**	≥0.05	≥0.05	**0.00**	**0.00**	**0.00**	**0.00**	-	≥0.05
TFBR-12	**0.00**	**0.00**	**0.00**	**0.00**	≥0.05	≥0.05	**0.00**	**0.00**	**0.00**	**0.00**	≥0.05	-

**Table 25:** Ranking of the best set based on the statistical test.

GWO		SCA		WOA	
set	Win	set	Win	set	Win
1. TFBR-5	8	1. TFBR-5	8	1. TFBR-5	8
2. TFBR-6	8	2. TFBR-6	8	2. TFBR-6	8
3. TFBR-11	8	3. TFBR-11	8	3. TFBR-11	8
4. TFBR-12	8	4. TFBR-12	8	4. TFBR-12	8
5. TFBR-1	5	5. TFBR-1	5	5. TFBR-1	5
6. TFBR-7	5	6. TFBR-7	5	6. TFBR-7	5
7. TFBR-10	5	7. TFBR-9	5	7. TFBR-9	5
8. TFBR-4	3	8. TFBR-3	4	8. TFBR-2	2
9. TFBR-9	3	9. TFBR-2	0	9. TFBR-3	2
10. TFBR-3	2	10. TFBR-4	0	10. TFBR-4	2
11. TFBR-8	1	11. TFBR-8	0	11. TFBR-8	0
12. TFBR-2	0	12. TFBR-10	0	12. TFBR-10	0

**Table 26:** Best sets of actions.

Set of Actions			
Set ID	Transfer Functions	Binarization Rules	Amount of actions
TFBR-5	S-shaped and V-shaped	Elitist	8
TFBR-6	S-shaped and V-shaped	Roulette Elitist	8
TFBR-11	S-shaped, V-shaped, X-shaped, and Z-shaped	Elitist	16
TFBR-12	S-shaped, V-shaped, X-shaped, and Z-shaped	Roulette Elitist	16

## Data Availability

All the results of this work are available at https://github.com/joselemusr/BSS-the-Transfer-Function-really-important (accessed on 23 May 2023).
